# Early experiences mediate distinct adult gene expression and reproductive programs in *Caenorhabditis elegans*

**DOI:** 10.1371/journal.pgen.1007219

**Published:** 2018-02-15

**Authors:** Maria C. Ow, Kirill Borziak, Alexandra M. Nichitean, Steve Dorus, Sarah E. Hall

**Affiliations:** 1 Department of Biology, Syracuse University, Syracuse, NY, United States of America; 2 Center for Reproductive Evolution, Department of Biology, Syracuse University, Syracuse, NY, United States of America; The University of North Carolina at Chapel Hill, UNITED STATES

## Abstract

Environmental stress during early development in animals can have profound effects on adult phenotypes via programmed changes in gene expression. Using the nematode *C*. *elegans*, we demonstrated previously that adults retain a cellular memory of their developmental experience that is manifested by differences in gene expression and life history traits; however, the sophistication of this system in response to different environmental stresses, and how it dictates phenotypic plasticity in adults that contribute to increased fitness in response to distinct environmental challenges, was unknown. Using transcriptional profiling, we show here that *C*. *elegans* adults indeed retain distinct cellular memories of different environmental conditions. We identified approximately 500 genes in adults that entered dauer due to starvation that exhibit significant opposite (“seesaw”) transcriptional phenotypes compared to adults that entered dauer due to crowding, and are distinct from animals that bypassed dauer. Moreover, we show that two-thirds of the genes in the genome experience a 2-fold or greater seesaw trend in gene expression, and based upon the direction of change, are enriched in large, tightly linked regions on different chromosomes. Importantly, these transcriptional programs correspond to significant changes in brood size depending on the experienced stress. In addition, we demonstrate that while the observed seesaw gene expression changes occur in both somatic and germline tissue, only starvation-induced changes require a functional GLP-4 protein necessary for germline development, and both programs require the Argonaute CSR-1. Thus, our results suggest that signaling between the soma and the germ line can generate phenotypic plasticity as a result of early environmental experience, and likely contribute to increased fitness in adverse conditions and the evolution of the *C*. *elegans* genome.

## Author summary

Environmental stress during early development in animals can have profound effects on adult behavior and physiology due to programmed changes in gene expression. However, whether different stresses result in distinct changes in traits that allow stressed animals to better survive and reproduce in future adverse conditions is largely unknown. Using the animal model system, *C*. *elegans*, we show that adults that experienced starvation exhibit opposite (“seesaw”) genome-wide gene expression changes compared to adults that experienced crowding, and are distinct from animals that experienced favorable conditions. Genes that are similarly up- or downregulated due to either starvation or crowding are located in clusters on the same chromosomes. Importantly, these gene expression changes of differently-stressed animals result in corresponding changes in progeny number, a life history trait of evolutionary significance. These distinct gene expression programs require different signaling pathways that communicate across somatic and germline tissue types. Thus, different environmental stresses experienced early in development induce distinct signaling mechanisms to result in changes in gene expression and reproduction in adults, and likely contribute to increased survival in future adverse conditions.

## Introduction

Phenotypic plasticity in response to environmental stress is a critical component of organismal fitness. Environmentally-induced phenotypic variation is thought to result, in part, from programmed changes in gene expression inherited through cell divisions or transgenerationally via epigenetic mechanisms such as DNA methylation, histone modifications, and non-coding RNAs [1]. For example, in nematodes, *Drosophila*, and humans, nutritional status during early development can modulate the longevity of subsequent generations in the absence of the original stimulus [2–4]. In *C*. *elegans*, the observed increased longevity is dependent upon the inheritance of starvation-induced non-coding small RNAs, likely imported into the germ line from the soma [3]. While this example is intriguing, an important and unresolved question is to what extent does environmentally-induced phenotypic plasticity mediated by changes in epigenetic marks result in adaptive variation of traits that is favored by natural selection [5, 6].

The free-living nematode *C*. *elegans* is an excellent model system to investigate the molecular mechanisms regulating environmentally induced phenotypic plasticity because its developmental trajectory is dependent on the environmental conditions experienced early in life. If conditions are favorable, *C*. *elegans* undergo continuous development consisting of four larval stages (L1-L4) followed by reproductive adulthood [7]. When faced with environmental stress (*e*.*g*. over-population, low food supply, or elevated temperatures), L1 larvae initiate an alternative diapause stage named dauer. Dauers are developmentally arrested, non-feeding, non-aging larva that exit diapause only if environmental conditions are favorable [8]. Natural populations of *C*. *elegans* experience a “boom and bust” reproductive strategy whereby they exist primarily as dauers for stress resistance and geographic dispersal but reproduce rapidly when food is available [9]. A potential prerequisite for the evolution of mechanisms that modulate adult phenotypes in response to specific stressors is that early environmental conditions are predictive of future conditions. The sensitivity of this developmental system to different dauer-inducing conditions, and how this may contribute to distinct phenotypic trajectories, is unknown. Our previous work has shown that postdauer (PD) adults that experienced crowding early in development exhibit changes in gene expression, genome-wide chromatin states, small RNA populations, and life history traits compared to isogenic animals that experienced continuous development (controls, CON) [10–12]. Thus, numerous molecular cues may have the potential to propagate information regarding early-life experiences to modulate adult developmental outcomes.

Over a century ago, August Weismann proposed in the germ-plasm theory of heredity that only germ cells, and not somatic cells, could pass heredity information [13]. Increasingly, studies have challenged the “Weismann Barrier” by demonstrating that the passage of non-coding RNAs between somatic tissues and the germ line can result in transgenerational inheritance [14]. For example, a recent report showed that *C*. *elegans* double-stranded RNA (dsRNA) generated in neurons and transported to the germ line resulted in transgenerational silencing that is dependent upon the main systemic RNA interference (RNAi) effector, SID-1 (WBGene00004795) [15]. Our previous work has shown that RNAi-pathways are required in different subsets of neurons for dauer formation in response to distinct environmental stresses, as well as the resulting reproductive plasticity observed between control and postdauer adults [11, 16]. These observations raised the intriguing possibility that postdauer animals that experienced different early life stresses retain distinct molecular signatures mediated by non-coding RNA signals.

Here, we investigate the effect that early environmental history has on modulating phenotypic plasticity in adults. We show that postdauer adults exposed to starvation (Stv) early in life exhibit distinct gene expression profiles and reproductive phenotypes when compared to postdauer adults that experienced crowding or high pheromone (Phe). These differences are highlighted by a set of “seesaw” genes that are oppositely regulated between postdauer and control adults depending on the experienced environmental stress. In addition, we provide evidence that the significant seesaw pattern of gene expression is due to RNAi-dependent fluctuations of gene expression across whole chromosomes, resulting in transcriptome-wide seesaw trends of gene expression changes in response to stress. Moreover, our results indicate that the distinct gene expression profiles in starvation versus pheromone conditions are dependent on germline- or somatic-generated signals, respectively, that potentially move between tissue types and mediate changes in brood size. Our results suggest a model where crosstalk between the soma and the germ line governs the mRNA transcriptome and reproductive plasticity following distinct environmental histories. Finally, we provide evidence that the relationship between early life environmental stress and distinct postdauer phenotypes has contributed to the evolution of *C*. *elegans* genome organization.

## Results

### Adult gene expression is dependent on environmental history

**Fig 1:**
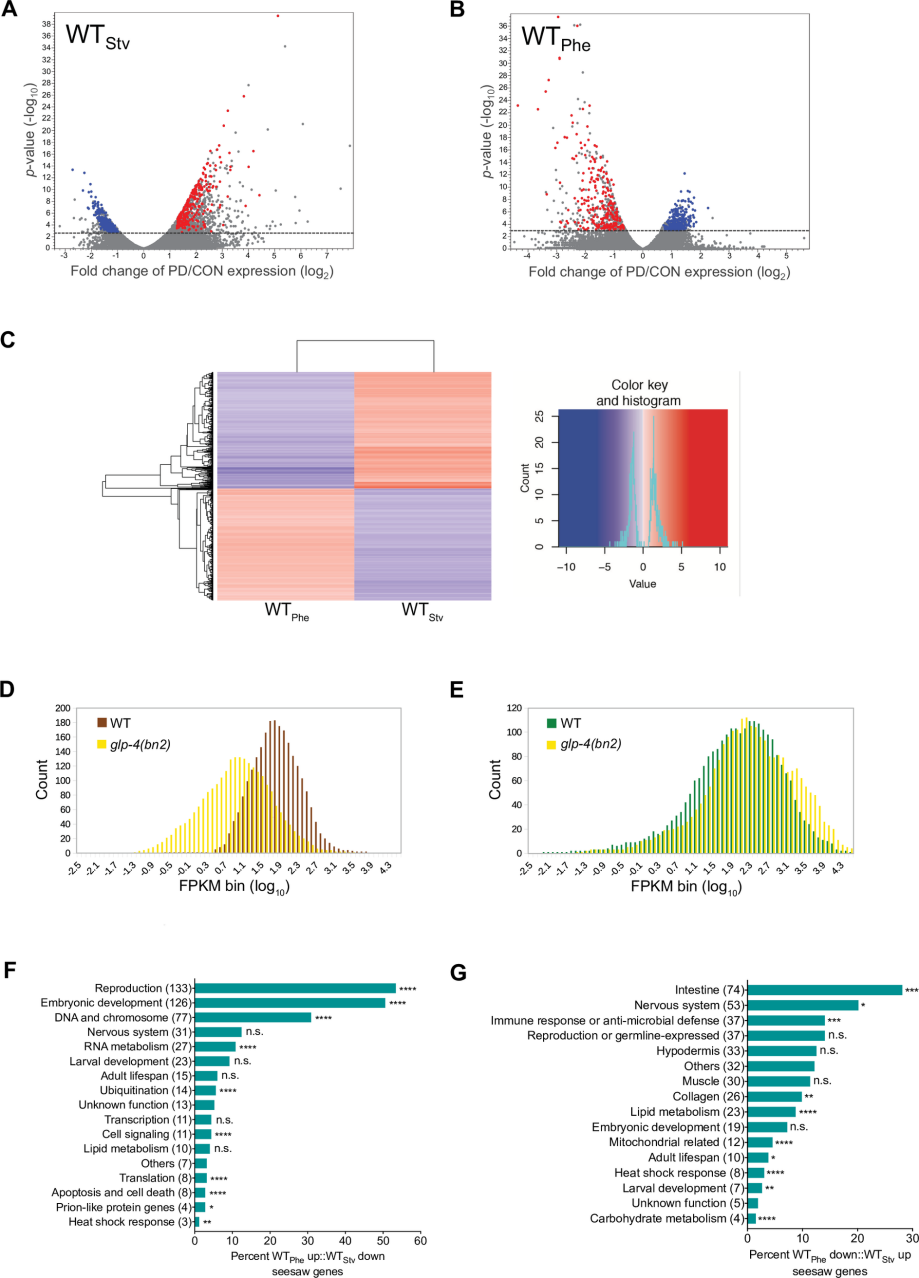
Early life history experience mediates gene expression in adults. (A, B) Volcano plots representing WT_Stv_ (PD_Stv_/CON_Stv_) and WT_Phe_ (PD_Phe_/CON_Phe_) RNA-Seq data. Dotted line indicates FDR cutoff of *p* = 0.05; dots above the lines are significantly different in PD compared to CON for a given condition. Red and blue dots represent the WT_Phe_ down::WT_Stv_ up and WT_Phe_ up::WT_Stv_ down seesaw genes, respectively. (C) Heat map depicting the expression levels of WT_Phe_ and WT_Stv_ seesaw genes. The complete list of seesaw genes can be found in S2 Table. A color key and histogram for the counts and values is shown in the right panel. (D, E) mRNA levels of (D) WT_Phe_ up::WT_Stv_ down and (E) WT_Phe_ down::WT_Stv_ up seesaw genes in wild-type and *glp-4(bn2)* strain [17]. The x-axis represents the fragments per kilobase of transcript per million mapped reads (FPKM) and the y-axis indicates the number of genes (count) per bin. (F, G) Putative function and spatial expression of the (F) WT_Phe_ up::WT_Stv_ down and (G) WT_Phe_ down::WT_Stv_ up seesaw genes. * *p* < 0.05; ** *p* < 0.01; *** *p* < 0.001; **** *p* < 0.0001; n.s. not significant (Fisher’s exact test) indicates enrichment compared to neutral expectations [20, 23–26, 83–85]. Number of genes for each category is in parentheses. See also S1, S2, S4 and S5 Tables.

To test the hypothesis that postdauer adult phenotypes are dependent upon the dauer-inducing stress experienced early in development, we conducted RNA-Seq using wild-type (WT) control (CON_Stv_) and postdauer adults that passed through the dauer stage as a result of having experienced starvation (PD_Stv_) (S1A–S1D and S2 Figs). Genes with significant changes in mRNA levels due to starvation-induced passage through the dauer stage were identified by comparing CON_Stv_ and PD_Stv_ libraries and subjected to a false discovery rate (FDR) *p*-value correction of less than 5%. We identified 1,121 and 551 genes that exhibited significant up- and downregulation, respectively, in wild-type PD_Stv_ compared to wild-type CON_Stv_ (WT_Stv_) (Fig 1A and S1 Table). To determine if PD_Stv_ adults are distinct from PD_Phe_, we repeated our previous experiment [10] and conducted RNA-Seq on CON_Phe_ and PD_Phe_ populations grown in parallel to the starvation samples. We identified 441 and 560 genes that were significantly up- or downregulated, respectively, in wild-type PD_Phe_ compared to wild-type CON_Phe_ (WT_Phe_) (Fig 1B and S1 Table).

Next, we compared the transcriptional changes between WT_Stv_ and WT_Phe_ to determine if different dauer-inducing stresses result in distinct transcriptional memories. A comparison of the 1,121 upregulated genes from WT_Stv_ to the 441 upregulated genes identified in WT_Phe_ identified only two genes (*y38f1a*.*6* (WBGene00012608) and *t05b11*.*3* (WBGene00020246)) in common. Likewise, when the 551 WT_Stv_ downregulated genes were compared to the 560 WT_Phe_ downregulated genes, there was no commonality (S3A Fig). However, we found that 249 (56%) of the 441 WT_Phe_ upregulated genes were downregulated in WT_Stv_ (WT_Phe_ up::WT_Stv_ down). Similarly, 263 (47%) of the 560 downregulated WT_Phe_ genes were upregulated in WT_Stv_ (WT_Phe_ down::WT_Stv_ up) (Figs 1A–1C and S3B and S2 Table). This observation indicates that not only do PD_Stv_ and PD_Phe_ adults have distinct transcriptional profiles, but also that a subset of genes is oppositely regulated based on the experienced dauer-inducing stress. Due to their propensity for being up- or downregulated in postdauers compared to controls depending on the animals’ environmental history, we refer to these 512 genes as the “seesaw” genes. Using random simulations, we determined that the observed numbers of seesaw genes in WT_Phe_ and WT_Stv_ gene sets are significantly greater than expected by chance (22.69-fold increase, *p* < 0.0001 and 9.38-fold increase, *p* < 0.0001, respectively).

To characterize the seesaw genes, we determined their expression patterns using several curation methods. First, we determined the enrichment of germline expressed seesaw genes by comparing their mRNA levels in wild-type animals to temperature-sensitive *glp-4(bn2)* (WBGene00006936) mutants [17]. At the restrictive temperature, *glp-4(bn2)* animals are deficient in germline stem cell proliferation, resulting in the lack of a functional germ line and sterility [18]. The analysis revealed that the expression level of WT_Phe_ up::WT_Stv_ down genes was substantially decreased in *glp-4(bn2)* compared to wild-type, indicating that these genes have germline enriched expression (Fig 1D). In contrast, WT_Phe_ down::WT_Stv_ up genes exhibited comparable expression levels in wild-type and *glp-4* germline-deficient worms (Fig 1E), suggesting that expression of these genes is enriched in somatic tissue. These trends in expression were further supported by examining the overlap of seesaw genes with tissue-enriched gene lists [19], which revealed that 73% of WT_Phe_ up::WT_Stv_ down seesaw genes were germline-enriched (S3C and S3D Fig and S3 Table). Lastly, we used the gene function descriptions in WormBase (WS253; [20]) consisting of published observations, GO annotations, tissue expression data, modENCODE [21], and Ensembl [22] information to further curate seesaw gene predicted function and spatial expression. Using these combined curation sources, we found an over-representation of genes associated with reproduction and embryonic development amongst the WT_Phe_ up::WT_Stv_ down seesaw genes [23, 24] (Fig 1F and S4 Table). In contrast, the expression of WT_Phe_ down::WT_Stv_ up seesaw genes are enriched in the intestine and nervous system, and have putative functions associated with innate immune response or anti-microbial defense [23–26] (Fig 1G and S5 Table). Together, these results indicate that *C*. *elegans* animals maintain a cellular memory of their early life experience through the expression of sets of genes that are sensitive to environmental history and are distinct in their expression profiles and functional composition.

### CSR-1 is required for gene expression changes between control and postdauer adults

**Fig 2:**
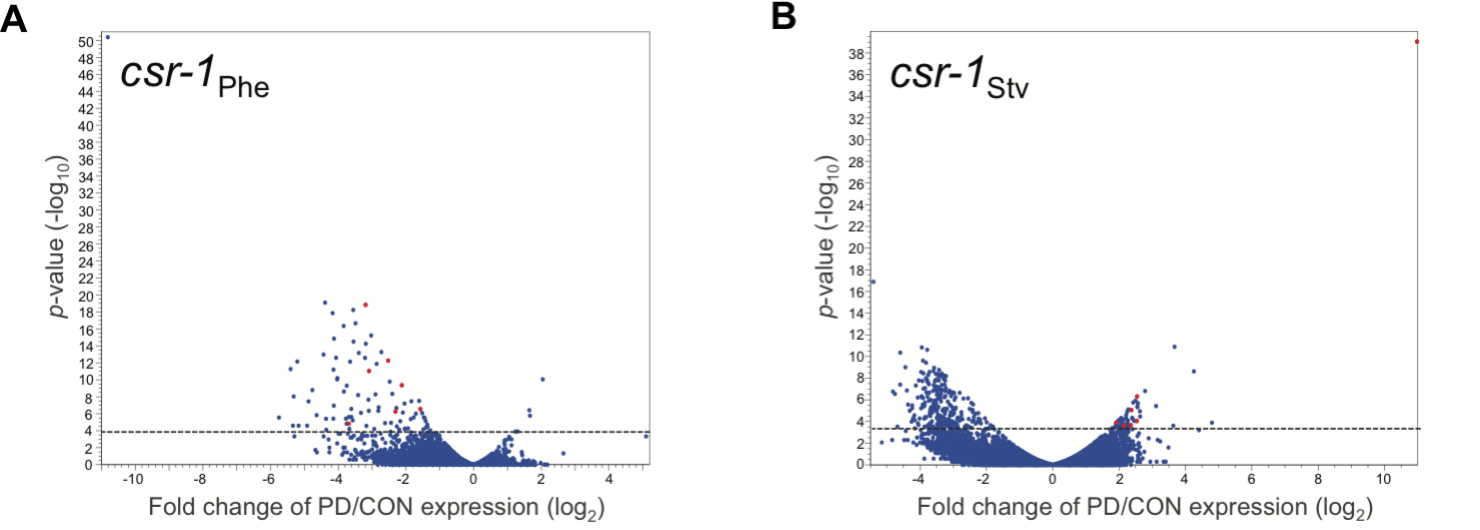
Environmentally programmed gene expression changes are dependent on CSR-1. (A, B) Volcano plots depicting *csr-1*_Phe_ (PD_Phe_/CON_Phe_) and *csr-1*_Stv_ (PD_Stv_/CON_Stv_) RNA-Seq data. Dotted line indicates FDR cutoff *p* = 0.05; dots above the lines are significantly different in PD compared to CON for a given condition. Red dots represent the *csr-1*_Phe_ down::*csr-1*_Stv_ up seesaw genes.

CSR-1 (WBGene00017641) is a *C*. *elegans* Argonaute protein that protects germline-expressed “self” transcripts from RNAi silencing through the organization of active chromatin domains and promotion of sense-oriented RNA polymerase II transcription genome-wide [27–32]. We previously showed that the CSR-1 RNAi pathway is required in early larval stages for dauer formation in response to starvation and high pheromone conditions and contributes to stable PD/CON changes in the chromatin state and gene expression for a subset of genes in adults [11, 16]. In addition, we found 95% of the WT_Phe_ up::WT_Stv_ down seesaw genes overlapped with a previously identified list of genes targeted by CSR-1 in the germ line [29], suggesting the possibility that CSR-1 may play a prominent role in the regulation of postdauer transcriptional memory as a consequence of environmental history. In contrast, only 1.1% of genes in the WT_Phe_ down::WT_Stv_ up dataset have been identified as CSR-1 targets. We therefore examined if the loss of CSR-1 affected the transcriptional changes observed in WT_Stv_ and WT_Phe_. Since *csr-1* null mutants are sterile, we used a *csr-1* hypomorph where sterility is partially rescued with a germline specific transgene [29], and performed RNA-Seq on *csr-1* control adults and starvation- and pheromone-induced *csr-1* postdauer adults (S1E–S1H and S2 Figs). Comparison of PD/CON gene expression levels for starvation and pheromone conditions (*csr-1*_Stv_ and *csr-1*_Phe_) revealed that 48 genes were significantly upregulated and 224 genes were downregulated in *csr-1*_Stv,_ while only 3 genes were significantly upregulated and 87 genes were downregulated in *csr-1*_Phe_ (Fig 2 and S1 Table). In addition, only 7 genes (*abu-14* (WBGene00004174), *c47f8*.*7* (WBGene00008163), *cut-2* (WBGene00009983), *f53a9*.*8* (WBGene00018731), *r02f11*.*1* (WBGene00019839), *tts-1* (WBGene00006650), and *tts-2* (WBGene00006651)) exhibited *csr-1*_Phe_ down::*csr-1*_Stv_ up seesaw patterns of gene expression, amongst which only one (*f53a9*.*8* (WBGene00018731)) was also found to seesaw in the WT_Phe_ down::WT_Stv_ up dataset. None of the genes exhibited a *csr-1*_Phe_ up::*csr-1*_Stv_ down seesaw expression pattern (Figs 2 and S3E, S3F and S2 Table). These results indicate that a functional CSR-1 RNAi pathway is required for the transcriptional memory of developmental history in starvation and high pheromone dauer-inducing conditions, including for the majority of WT_Phe_ down::WT_Stv_ up genes that have not been previously identified as CSR-1 targets.

**Fig 3:**
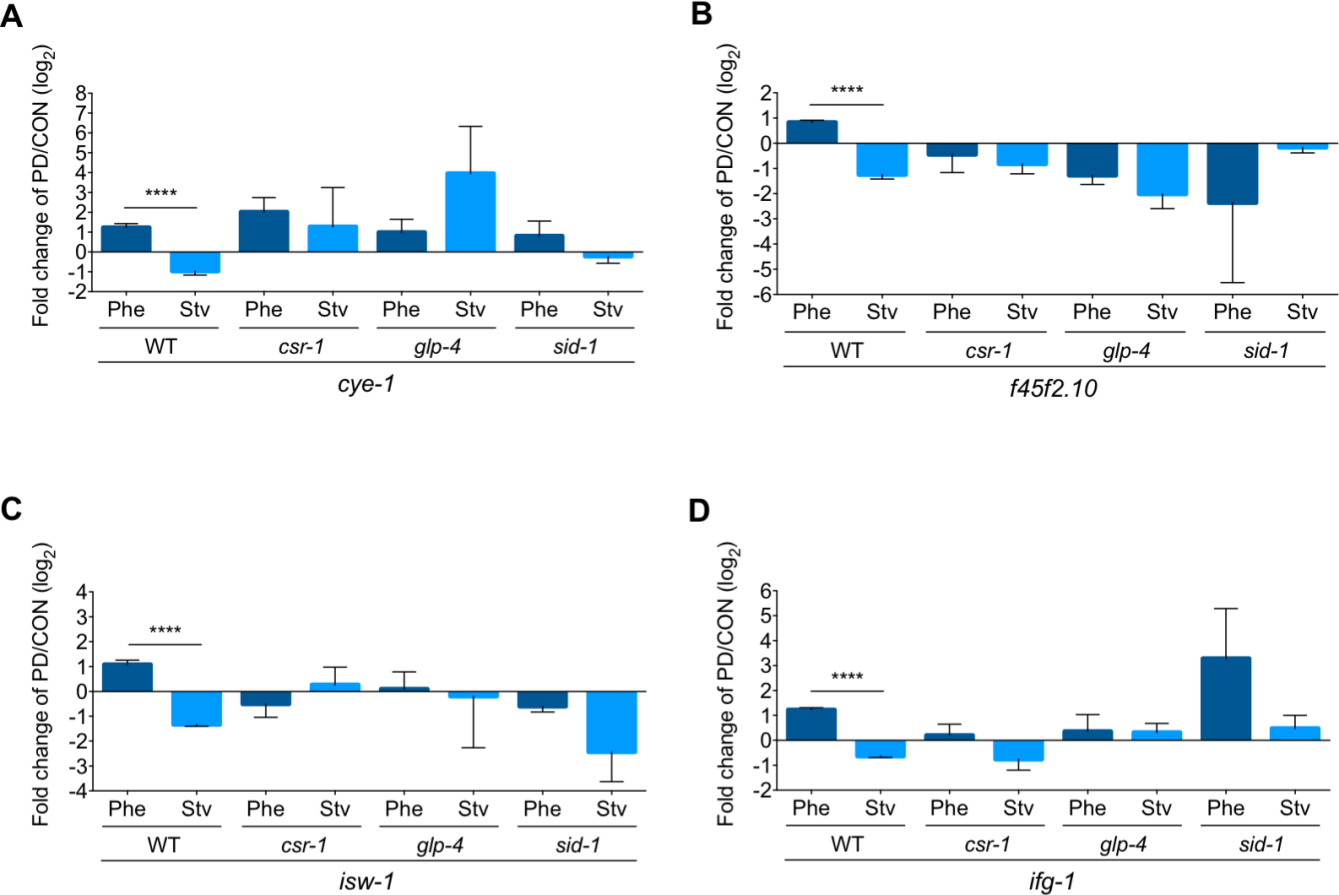
SID-1 and CSR-1 are required for seesaw gene expression changes in the germ line. (A-D) qRT-PCR validation of germline-expressed, CSR-1-targeted seesaw gene PD/CON mRNA levels in WT, *csr-1* hypomorph, *glp-4(bn2*) (grown at 25°C), and *sid-1(qt9)* strains for Phe and Stv conditions. **** *p* < 0.0001; Student’s *t*-test comparison of Phe (PD_Phe_/CON_Phe_) and Stv (PD_Stv_/CON_Stv_) within a strain. Error bars represent Standard Error of the Mean (S.E.M.). qRT-PCR validations for additional genes are found in S3, S4, S5, and S6 Figs.

We sought to verify the RNA-Seq results using qRT-PCR on a subset of genes from biologically independent samples of wild-type and *csr-1* hypomorph strains. First, we examined the mRNA levels of 12 germline-specific, CSR-1-targeted genes that exhibited the WT_Phe_ up::WT_Stv_ down seesaw pattern. Seven (*cye-1* (WBGene00000871), *f45f2*.*10* (WBGene00018482), *isw-1* (WBGene00002169), *ifg-1* (WBGene00002066), *cbd-1* (WBGene00010351), *daz-1* (WBGene00000935), and *lin-41* (WBGene00003026)) of the 12 genes (58%) were validated in wild-type animals (Figs 3, S4C and S4E); however, none of the wild-type seesaw patterns were validated in the *csr-1* hypomorph (Figs 3 and S5C). For the validated genes, the abrogation of seesaw gene expression in the *csr-1* hypomorph was due to multiple effects on gene expression levels in postdauers and/or controls, including: PD/CON direction of gene expression change inverting in both conditions (29%); both conditions exhibiting similar direction of change to either WT_Phe_ (29%) or WT_Stv_ (29%); or one or both conditions no longer exhibiting a significant change in PD/CON mRNA levels (14%) (S4C and S5C Figs).

**Fig 4:**
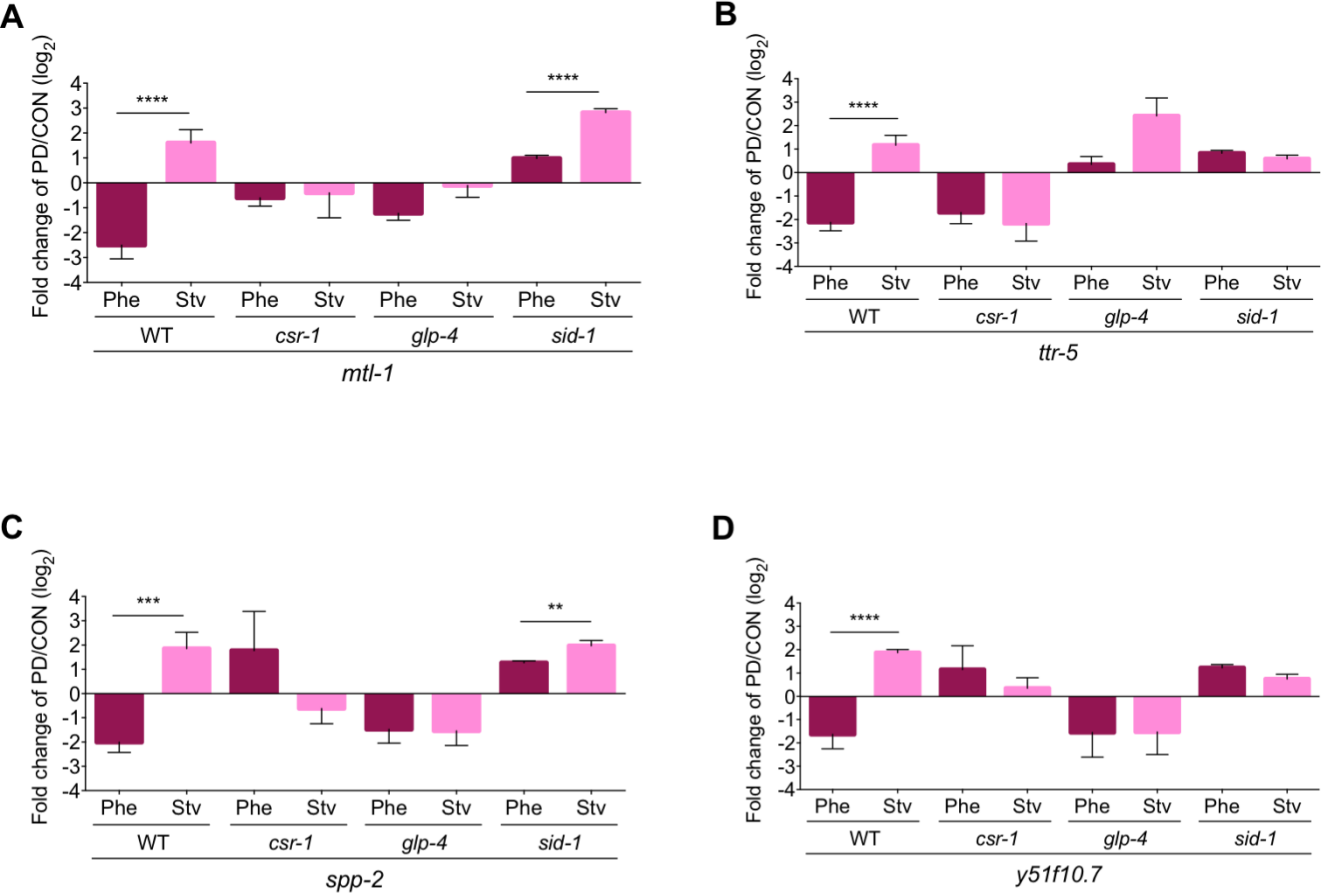
The germ line, SID-1, and CSR-1 are required for seesaw gene expression changes in the soma. (A-D) qRT-PCR validation of mRNA levels of soma-expressed, non-CSR-1 targeted seesaw gene PD/CON mRNA levels in WT, *csr-1* hypomorph, *glp-4(bn2)* (grown at 25°C), and *sid-1(qt9)* strains for Phe and Stv conditions. ** *p* < 0.01, *** *p* < 0.001, **** *p* < 0.0001; Student’s *t*-test comparison of Phe (PD_Phe_/CON_Phe_) and Stv (PD_Stv_/CON_Stv_) within a strain. Error bars represent S.E.M. qRT-PCR validations for additional genes are found in S3, S4, S5 and S6 Figs.

Next, we sought to validate the mRNA levels of 12 soma-enriched, non-CSR-1-targeted genes that exhibited the WT_Phe_ down::WT_Stv_ up seesaw patterns. We validated 10 (*ins-19* (WBGene00002102), *mtl-1* (WBGene00003473), *f55b11*.*4* (WBGene00010086), *ttr-5* (WBGene0000804), *fmi-1* (WBGene00001475), *hsp-16*.*41* (WBGene00002018), *f53a9*.*8* (WBGene00018731), *r12e2*.*15* (WBGene00020040), *spp-2* (WBGene00004987), and *y51f10*.*7* (WBGene00021768)) out of the 12 genes (83%) in wild-type samples (Figs 4, S4B and S4E). Consistent with our transcriptome data, the seesaw gene expression pattern for a majority of these genes were also dependent on CSR-1, despite not being previously identified as targets of CSR-1 nor being germline-enriched. Only 2 genes, *hsp-16*.*41* and *r12e2*.*15*, retained a significant seesaw pattern in the *csr-1* hypomorph; however, in both cases, the change in expression between *csr-1*_Phe_ and *csr-1*_Stv_ is opposite to the change observed in wild-type samples (S4B and S5B Figs). For the remainder of the validated genes, they showed similar disruptions in expression in *csr-1* hypomorph compared to wild-type as the germline-enriched genes: the PD/CON direction of gene expression inverting in both conditions (30%); both conditions exhibited trends in gene expression similar to either WT_Phe_ (20%) or WT_Stv_ (10%); or that one or both conditions no longer exhibited a significant change in PD/CON mRNA levels (20%). These results further confirm that CSR-1 plays a crucial role in mediating the PD/CON gene expression changes based on environmental history, regardless of whether the gene is a known CSR-1 target or not.

### SID-1 requirement for seesaw gene expression

Thus far, our implication of the CSR-1 RNAi pathway in the regulation of soma-enriched, non-CSR-1 target seesaw genes suggests the possibility that signals transported between cell and tissue types could contribute to the transcriptional memory of environmental history. In *C*. *elegans*, systemic RNAi spreads dsRNA throughout the animal and requires its main effector, the dsRNA importer, SID-1 [33, 34]. To ascertain whether the transport of dsRNA is a mechanism eliciting the starvation- and pheromone-induced seesaw effect, we measured the PD/CON mRNA levels of genes in the *sid-1(qt9)* null mutant. Similar to the *csr-1* hypomorph, both germline- and soma-enriched genes failed to exhibit a seesaw pattern in *sid-1* adults (Figs 3, 4, S4 and S7). When we examined whether *sid-1* was required for the seesaw gene expression for a specific dauer-inducing condition, we found that 47% of the validated genes exhibited the opposite direction of change in *sid-1* compared to wild-type for the pheromone condition, compared to 20% showing a similar effect in starvation condition. Thus, SID-1 primarily contributes to seesaw gene expression profiles in somatic and germline tissues due to early life history of the pheromone condition.

### Germline requirement for seesaw gene expression

The observation that signals from the germ line can mediate somatic gene expression levels to affect adult lifespan is well-established in *C*. *elegans*, *Drosophila*, and mammals [35]. Since we have shown that the systemic RNAi effector, SID-1, is playing a role in the transcriptional memory of environmental history, we interrogated whether signals exported from the germ line are necessary for the seesaw pattern in adult somatic tissue. To examine the potential role of the germ line in modulating the gene expression changes due to environmental history, we performed qRT-PCR to measure PD/CON mRNA levels for germline- and soma-enriched seesaw genes in a strain carrying the *glp-4(bn2)* allele, which lacks a functional germ line at the restrictive temperature [18]. As expected in animals lacking a germ line, the pheromone- and starvation-induced seesaw expression of the 12 germline-enriched genes was abolished in *glp-4(bn2)* adults grown at the restrictive temperature (Figs 3 and S6C). Similarly, all but one (*f55b11*.*4*) of the 13 soma-enriched genes also showed an elimination of the seesaw pattern in *glp-4(bn2)* adults (S6B Fig). These results indicate that the seesaw pattern of gene expression, including genes that have enriched expression in the soma, requires a functional germ line. Again, to examine whether the germ line is required for the regulation of gene expression in specific dauer-inducing conditions, we examined whether PD/CON ratio of mRNA levels were affected for the pheromone or starvation conditions in *glp-4* animals. For both germline and soma-enriched genes, we observed that 71% of the validated genes exhibited the opposite direction of change in expression in *glp-4* compared to wild-type for the starvation condition, while only 24% exhibited this effect for the pheromone condition. Thus, a functional germ line is paramount for the programmed change in PD/CON mRNA levels in both the soma and germ line as a result of early life starvation. Furthermore, since SID-1 is not required for starvation-induced expression changes, these results indicate that the germline-dependent signal regulating somatic gene expression is not dsRNA.

### Genome-wide seesaw patterns are chromosome dependent

**Fig 5:**
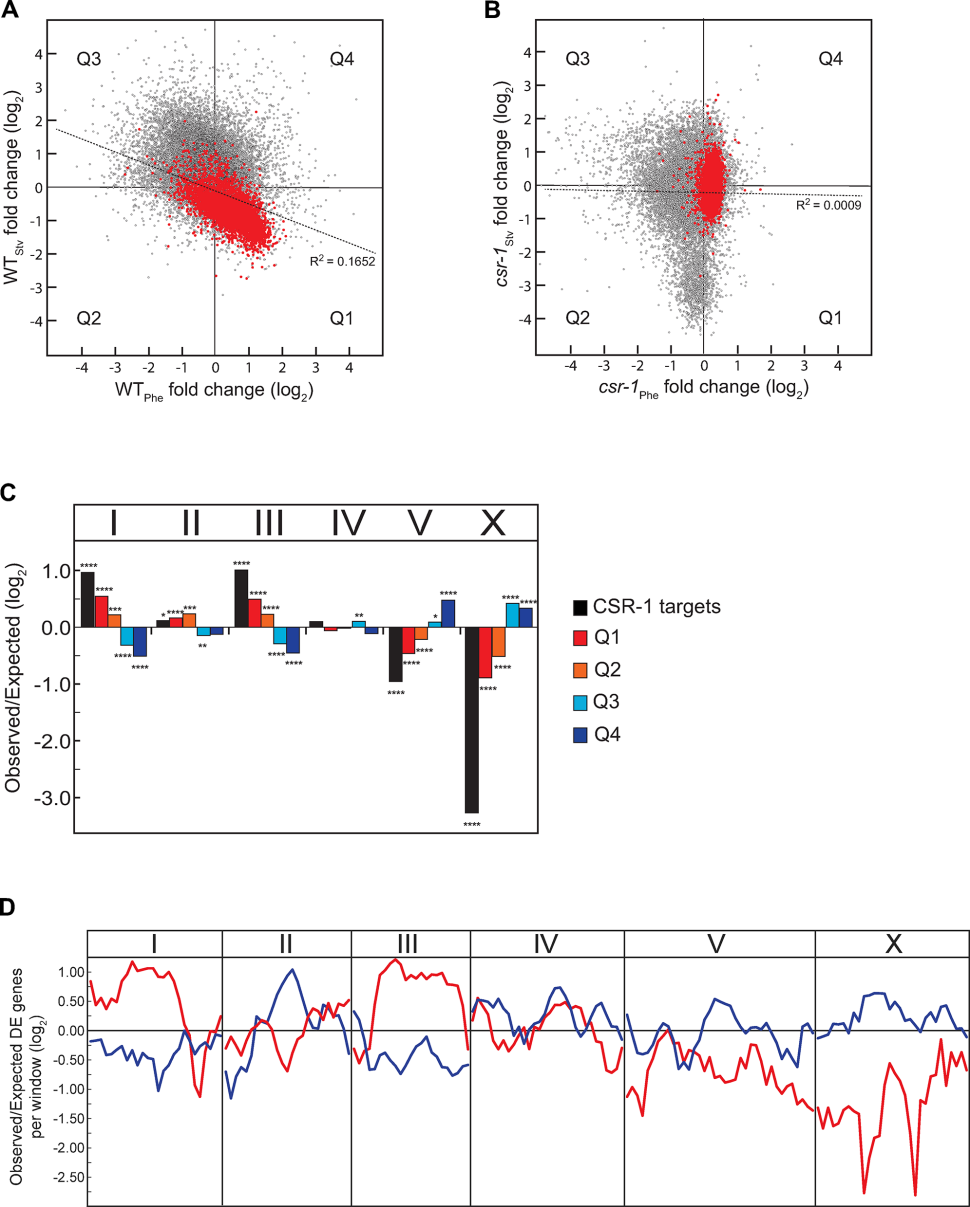
Seesaw changes of gene expression occur genome-wide. (A, B) PD/CON mRNA levels of all genes using the log_2_ transformed EdgeR fold change values in (A) WT_Phe_ and WT_Stv_ or (B) *csr-1*_Phe_ and *csr-1*_Stv_. CSR-1 targets are indicated in red. Significance of correlation was calculated based on the Pearson's correlation between samples. Genes are categorized by quadrant that correspond to their PD/CON mRNA levels in Phe and Stv conditions. (C) Chromosomal bias of CSR-1 targets and expression changes for all genes. Genes are categorized by quadrant as shown in Fig 5A. * *p* < 0.05; ** *p* < 0.001; *** *p* < 0.001; **** *p* < 0.0001; χ^2^comparison to uniform distribution based on gene number per chromosome. (D) Sliding window analyses of physical distribution of genes with a 2-fold and greater change in PD/CON mRNA levels for WT_Phe_ up::WT_Stv_ down (red line) and WT_Phe_ down::WT_Stv_ up (blue line) genes relative to a uniform distribution across the genome.

The significant excess of genes exhibiting seesaw patterns of differential expression and the ability of CSR-1 to modulate expression of non-target genes led us to investigate whether the inverse expression response to distinct dauer-inducing stresses may be a genome-wide phenomenon. This analysis revealed that a large proportion of genes whose PD/CON mRNA levels were not significantly seesawing by our original, stricter criteria still exhibited opposite changes in PD/CON mRNA levels with respect to the dauer-inducing stress. A significant inverse correlation in gene expression change was observed for 67.1% of genes (12,454 out of 18,570 genes sampled in both experiments) in response to two dauer triggers (R^2^ = 0.167, *p* < 0.0001) (Fig 5A, Q1 and Q3). Consistent with our previous analysis, most CSR-1 targets (74.3%) exhibited the WT_Phe_ up::WT_Stv_ down pattern of expression (Fig 5A, Q1). Moreover, when we compared the transcriptome of *csr-1*_Phe_ to *csr-1*_Stv_, we observed a pattern distinct from wild-type, particularly for genes that upregulated in WT_Phe_ dataset (Fig 5A and 5B, Q1 and Q4). This analysis suggests that a majority of the genes in the genome, in addition to our identified set of seesaw genes, are subject to trends of CSR-1 dependent differential regulation in response to environmental history.

A recent report found that genes that were similarly downregulated via RNAi-mediated chromatin remodeling in *S*. *pombe* during quiescence are located in clusters throughout the genome [36]. In *C*. *elegans*, thousands of protein-coding, germline-expressed genes are physically clustered in euchromatic domains that are established and maintained by the CSR-1 pathway [29, 31, 32]. To assess whether the physical location of *C*. *elegans* genes correlates with the observed transcriptome-wide seesaw patterns of gene regulation, we examined the location of CSR-1 target genes throughout the genome. First, examination of the chromosomal distribution of CSR-1 targets identified a highly significant enrichment on chromosomes I and III and paucity on chromosome V and sex chromosome X (Fig 5C). Second, a striking bias that parallels the distribution of CSR-1 targets was observed for the genomic distribution of genes based on their response to the starvation condition. Genes that were downregulated in WT_Stv_ (Q1 and Q2) were overrepresented on chromosomes I and III, and genes that are upregulated in WT_Stv_ (Q3 and Q4) are overrepresented on chromosomes V and X (Fig 5A and 5C). Expression patterns of genes in the pheromone condition did not correlate with their distribution across chromosomes beyond the seesaw relationship in expression responses observed in Q1 and Q3 (Fig 5A). Thus, our analysis shows that genes with similar trends in expression patterns in starvation condition are non-randomly distributed across chromosomes, with CSR-1 targets overrepresented on the same chromosomes as genes that are downregulated in WT_Stv_.

In light of the marked chromosomal bias in expression patterns of genes in response to environmental history, we next investigated whether genes with a specific expression pattern exhibited spatial organization within chromosomes. Low recombination in the center of *C*. *elegans* chromosomes has resulted in extensive linkage disequilibrium and the operation of selection at the level of large haplotype blocks [37], possibly facilitating the distribution of similarly regulated genes in clusters. A sliding window approach was used to assess the distribution of inversely regulated genes (Q1 and Q3) exhibiting at least 2-fold change in PD/CON mRNA levels for pheromone and starvation conditions. This analysis confirmed a general enrichment of Q1 genes (red lines) in central regions of chromosomes I and III, and Q3 genes (blue lines) in the central regions of chromosomes II, V, and X (Fig 5D). Further, to define the location of CSR-1 target genes relative to each other, we employed a genome-wide clustering algorithm using 4,191 CSR-1 targets identified in the germ line [29] in order to delineate CSR-1 “clusters.” We found that 73% of CSR-1 targets mapped to 507 clusters ranging from 3 to 77 genes, with most (78%) being between 3 to 10 genes long (S6 Table). The number of clusters (1.33x; *p* < 0.0001) and the number of clustered CSR-1 targets (1.73x; *p* < 0.0001) both exceeded neutral expectations as defined by randomized gene order simulations. As would be expected, there was a highly significant enrichment of CSR-1 clusters on chromosomes I and III (*p* = 0.0002). Since germline-expressed genes and CSR-1 targets are enriched in operons [38, 39], we also examined the expression patterns of a defined set of *C*. *elegans* operons [40]. Interestingly, while 359 of the 901 known operons exhibit a WT_Phe_ up::WT_Stv_ down directional change in gene expression, only 91 of the 512 (18%) significant seesaw genes reside in operons, suggesting that operons alone do not account for our overarching genomic trends in gene expression. Thus, the co-localization of genes exhibiting similar seesaw trends in expression to particular chromosomes indicates that many of these loci would be simultaneously captured via genetic hitchhiking in the repeated selective sweeps that have shaped the *C*. *elegans* genome.

### Early life history mediates reproductive plasticity

**Fig 6:**
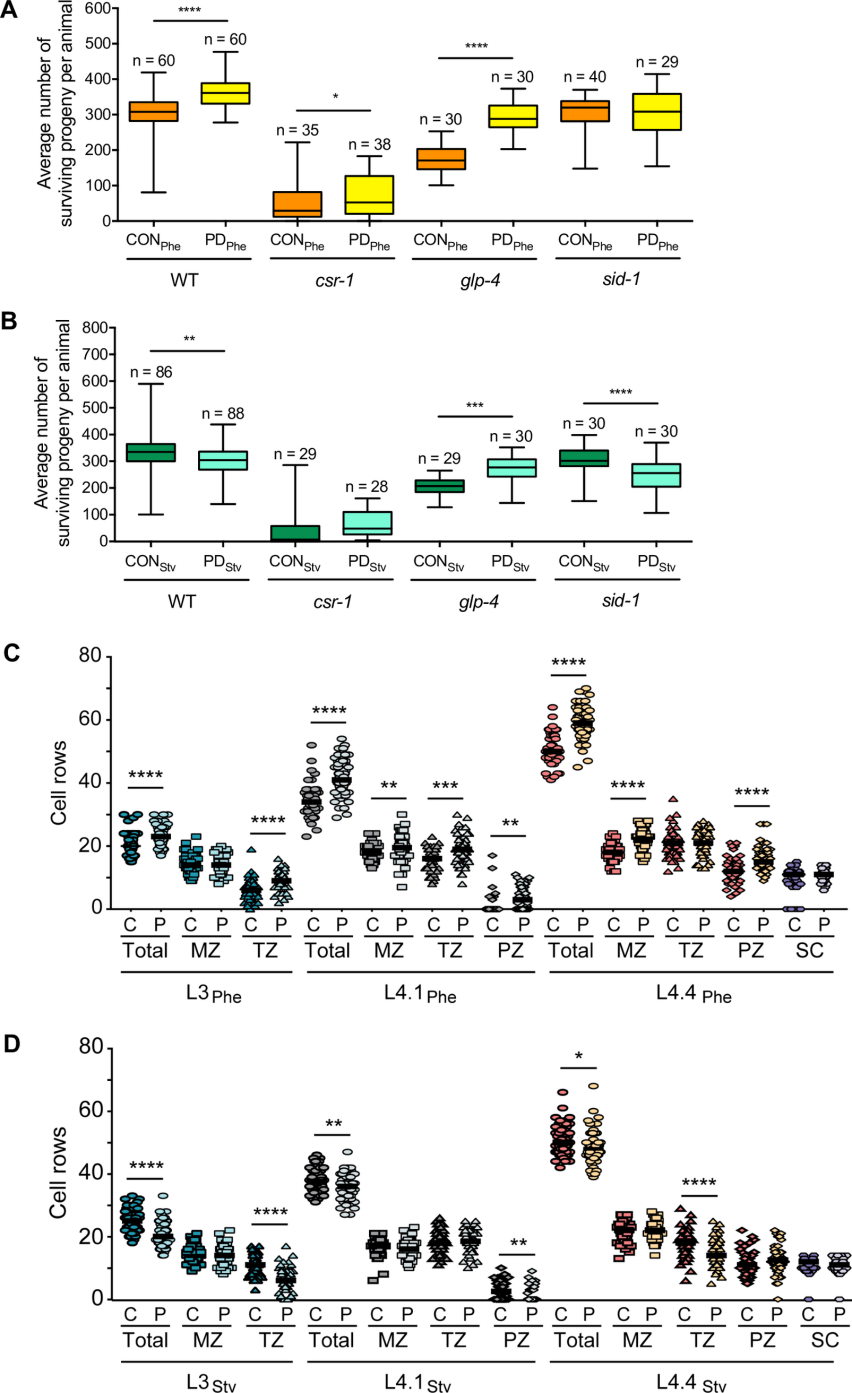
Reproductive plasticity is dependent on early life history. (A, B) Whisker-box plots (minimum and maximum of all data) of the average brood size for CON and PD that experienced (A) Phe or (B) Stv for WT, *csr-1* hypomorph, *glp-4(bn2)*, and *sid-1(qt9)* strains. Assays for WT, the *csr-1* hypomorph, and *sid-1(qt9)* were performed at 20°C; brood assays for *glp-4(bn2)* were performed at 15°C. Total sample number is indicated by n over at least 3 biologically independent trials. (C, D) Number of germ cell rows in one gonad arm of CON and PD larva that exhibit L3, L4.1, and L4.4 vulva morphology and experienced (C) Phe or (D) Stv conditions. MZ, mitotic proliferative zone; TZ, transition zone; PZ, pachytene zone; SC, spermathecal cells. N ≥ 45 animals over 3 biologically independent trials. * *p* < 0.05, ** *p* < 0.01, *** *p* < 0.001, **** *p* < 0.0001; Student’s *t*-test.

We next questioned whether the significant seesaw changes in mRNA levels due to environmental history result in phenotypic consequences in adult animals. Due to the overlap between germline-specific genes and WT_Phe_ up::WT_Stv_ down seesaw genes (Figs 1D, 1F and S3D), we hypothesized that a reproductive phenotype could be an outcome of an animal’s environmental history. To determine whether seesaw gene expression affected the number of progeny produced by wild-type postdauer hermaphrodites, we quantified the brood size of control and postdauer adults that experienced either pheromone or starvation. Consistent with our previous reports, wild-type PD_Phe_ had an increased brood size compared to wild-type CON_Phe_ (Fig 6A; [10, 11]). In contrast, wild-type PD_Stv_ had a reduced brood size compared to wild-type CON_Stv_ (Fig 6B). This indicates that the gene expression changes resulting from environmental history have significant consequences with respect to the fitness of *C*. *elegans* animals.

Since we observed that seesaw changes in gene expression are dependent on mechanisms involving CSR-1, GLP-4, and SID-1 functions (Figs 3, 4, S4, S5, S6 and S7), we asked whether the same mechanisms affected the fecundity differences observed in pheromone- or starvation-induced wild-type postdauer and control adults. Even with a reduced brood size, the number of surviving progeny produced by the *csr-1* hypomorph remained slightly higher in PD_Phe_ compared to CON_Phe_ (Fig 6A). However, the decrease in brood size between postdauer and control adults was abrogated in *csr-1*_Stv_ (Fig 6B). We also observed that *glp-4(bn2)* adults grown at the permissive temperature no longer exhibited a decrease in postdauer brood size in the starvation condition (Fig 6B), but continued to show an increased number of progeny in the pheromone condition (Fig 6A). Recently, GLP-4 was shown to be expressed in the intestine and somatic gonad in addition to the germ line [41]; thus, our observed *glp-4* PD/CON brood size phenotype at the permissive temperature suggests that GLP-4 function in the intestine or somatic gonad is contributing to the regulation of the starvation program (see Discussion).

In contrast, the brood size results of the *sid-1(qt9)* strain were opposite to those of the *glp-4(bn2)* strain. The increase in PD_Phe_/CON_Phe_ brood size observed for wild-type adults was abolished in *sid-1(qt9)* adults (Fig 6A); however, the decrease in PD_Stv_/CON_Stv_ brood size was also observed in *sid-1* adults (Fig 6B). Together, these results are in accordance with our gene expression analyses where changes in mRNA levels and the resulting phenotypic plasticity due to pheromone conditions are a result of SID-1 function in the soma, whereas starvation-induced changes are mediated by unknown signals from the germ line.

Next, we sought to further characterize the developmental differences in the germ line that could result in altered PD/CON brood sizes. Since reproduction in self-fertilizing *C*. *elegans* hermaphrodites is sperm-limited [42], we asked whether the fecundity differences in PD/CON was associated with changes in mRNA levels of genes regulating germ line mitotic proliferation and the onset of meiosis during spermatogenesis. Hermaphrodites possess two gonad arms, each of which is capped by a distal tip cell (DTC) that maintains the germline stem cell niche through GLP-1/Notch (WBGene00001609) signaling (mitotic zone, MZ) [43, 44]. As cells divide in the mitotic proliferative zone, the most proximal cells begin to express the RNA binding protein, GLD-1 (WBGene00001595), which promotes entry into meiosis (transition zone, TZ) [45, 46]. Since hermaphrodites produce all their sperm during the larval L4 stage, we hypothesized that modulation of these genes as a result of environmental history could potentially alter the number of sperm in hermaphrodite animals. To test our hypothesis, we first examined the expression of WT_Phe_ up::WT_Stv_ down seesaw gene, *gld-1*, using a *gld-1*::*gfp* transgene expressed in the germ line. To compare developmentally synchronized animals, we examined GFP levels of postdauer and control animals that experienced either pheromone or starvation conditions and exhibited the vulva morphology characteristic of L3, L4.1, and L4.4 larval animals [47, 48], at which times the mitotic and transition zones are evident [43]. Although we were unable to validate the seesaw changes in *gld-1* expression using qRT-PCR (S4C Fig), we observed that GFP levels in the germ line were significantly increased in PD_Phe_ larva compared to CON_Phe_ larva at all stages, consistent with our RNA-Seq results (S8A and S8B Fig and S11 Table). In contrast, we detected no significant change in GFP levels in PD_Stv_ compared to CON_Stv_ in animals exhibiting L3 vulval morphology, but a surprising increase in GFP levels in PD_Stv_ compared to CON_Stv_ for the L4.1 and L4.1 stages similar to the pheromone condition (S8A and S8C Fig and S11 Table). In Notch signaling mutants, GLD-1 levels remain stable in the transition zone [49], and high levels of GLD-1 are sufficient to drive germline stem cells into meiosis, even in the presence of Notch signaling [50]. Furthermore, we observed that the area of the gonad arms was also significantly different due to environmental history, such that postdauer gonad arm area was increased or decreased compared to controls in pheromone and starvation conditions, respectively, for all larval stages (S8B and S8C Fig and S11 Table). Together, these results suggest that the changes in GLD-1 levels we observed in Phe larva likely reflects alterations in the numbers of cells entering meiosis and not changes in GLD-1 expression in individual cells.

To further test our hypothesis, we DAPI-stained larva and counted the number of cell rows per gonad arm in control and postdauer animals that exhibited the characteristic L3, L4.1, and L4.4 vulva morphology and experienced either pheromone or starvation conditions (S9 and S10 Figs) [48, 51]. If the sperm to oocyte developmental switch remains constant between postdauer and control animals [52], we would predict that postdauer larva that experienced pheromone or starvation conditions to begin germline proliferation earlier or later than control larva, respectively, resulting in altered numbers of sperm available for self-fertilization in adult hermaphrodites. Indeed, we observed a significant increase for PD_Phe_/CON_Phe_ total cell rows and decrease for PD_Stv_/CON_Stv_ total cell rows for all developmental stages examined (Fig 6C and 6D and S12 Table). Interestingly, at the L3 stage, we observed a similar number of cell rows in the mitotic zone, but different numbers of cell rows in the transition zone for postdauers compared to controls in both conditions, suggesting that proliferation begins earlier or later in postdauer animals that experienced pheromone or starvation conditions, respectively (Fig 6C and 6D and S12 Table). As the animals aged and germ lines expanded, we observed that the numbers of cell rows in a particular region of the gonad, such as the transition zone or pachytene zone, varied between postdauer and control animals differently depending on the stage (L4.1 or L4.4) and environmental condition (pheromone or starvation). This result likely reflects the different mechanisms regulating pheromone and starvation gene expression changes. As an additional control, we also counted the number of cells in the spermatheca [53], which is a part of the somatic gonad, and found that the number of cells are similar for all the L4.4 populations as expected (Fig 6C and 6D and S12 Table). This result indicates that germline development, and the onset of germline proliferation, can be uncoupled from somatic gonad development, resulting in postdauer germ lines that are “older” or “younger” compared to their control counterparts. Based on our RNA-Seq data in adults (Fig 1A and 1B), these developmental trends seem to persist from the L3 stage into adulthood to result in the WT_Phe_ up::WT_Stv_ changes in expression of germline-enriched genes. Together, these results are consistent with the model that the onset of germline proliferation during L3 larval stages is determined by environmental and developmental history, resulting in altered sperm number and brood size in adults.

Furthermore, we sought to identify genes in addition to *gld-1* that may contribute to altered germline development and spermatogenesis due to environmental experience. Using sperm transcriptome and proteome datasets [54], we found significant overlaps between the sperm transcriptome and proteome with the WT_Phe_ up:: WT_Stv_ down (*p* < 0.0001 and *p* = 0.002, respectively; two-tailed Fisher’s exact test) and WT_Phe_ down:: WT_Stv_ up (*p* < 0.0001 and *p* < 0.0001, respectively; two-tailed Fisher’s exact test) seesaw genes. GO term analyses revealed significant functional distinctions between the genes encoding protein components of sperm (“sperm genes”) overlapping with the two classes of seesaw genes. Sperm genes associated with the WT_Phe_ up:: WT_Stv_ down seesaw genes were enriched for reproduction, genitalia development, oogenesis, and spermatogenesis, while the sperm genes overlapping with the WT_Phe_ down:: WT_Stv_ up seesaw genes were devoid of reproduction-related functions and were instead richly affiliated with the cuticle and collagen (S7 Table). Additional experimentation will be required to determine if the changes in sperm genes are causal to, or result from, changes in germline development in larva. In sum, these results indicate that the fecundity differences resulting from distinct life histories may have a direct relationship with the differential expression of sperm-related genes with diverging functionalities.

**Fig 7:**
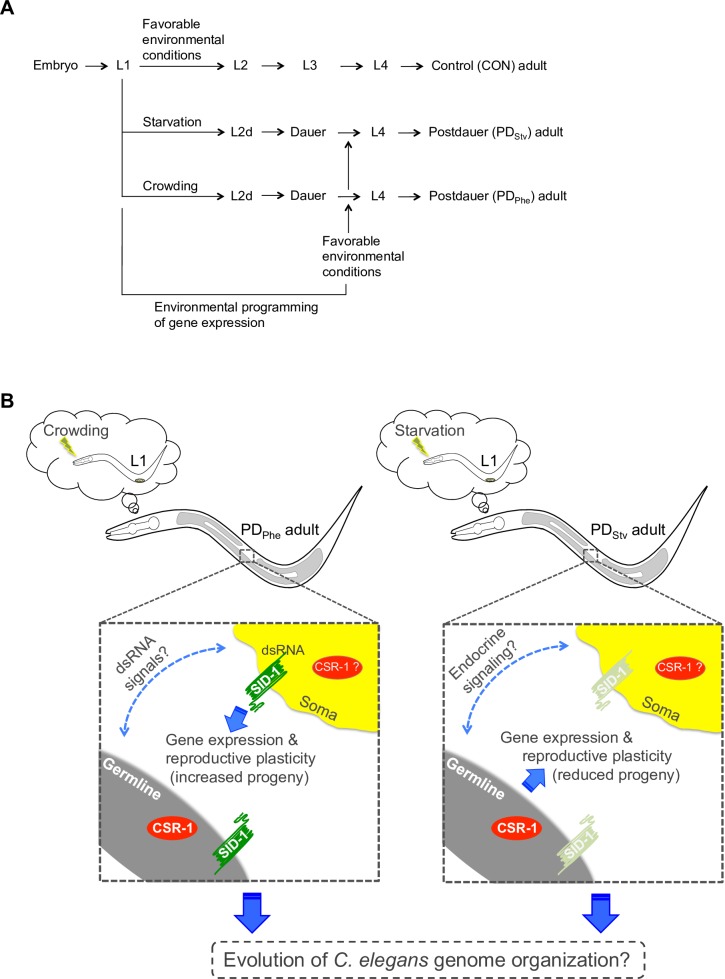
Distinct early life histories result in different gene expression programs and reproductive plasticity in adults. (A) *C*. *elegans* life history trajectory is dependent on the environmental conditions experienced during L1 larval stage. CON refers to control adults that did not experience environmental stress. Postdauer adults transiently passed through the dauer stage due to starvation (PD_Stv_) or overcrowding (PD_Phe_) conditions. (B) PD animals retain a cellular memory of early environmental history that governs gene expression and reproductive plasticity. The germ line, SID-1, and CSR-1 mediate the Phe and Stv programs. See text for details.

Taken together, we posit a model whereby different “programs” regulate global changes in gene expression leading to distinct reproductive phenotypes. In animals that experience early life high pheromone condition, the changes in PD/CON mRNA levels in the germ line and soma are dependent on SID-1 function in the soma. In contrast, in postdauer animals that experience early life starvation condition, the observed gene expression changes in the germline and the soma are not dependent on SID-1, but are instead dependent on an unidentified signal(s) from a functional germ line. Our data indicate that these two programs maintain a functional balance within the animal, such that when one program is disrupted by mutation (e.g. *glp-4(bn2)* mutant), the animal exhibits the gene expression and reproductive phenotype of the alternate program (Figs 3, 4, 6, S4, S5, S6 and S7). In addition, both programs are dependent on the CSR-1 RNAi pathway for these chromosomally-regulated gene expression differences to result in reproductive plasticity of adult animals (Fig 7). Moreover, we provide evidence that genes with similar trends in expression levels in response to pheromone and starvation conditions are co-localized on specific chromosomes, and may have contributed to the evolution of the *C*. *elegans* genome.

## Discussion

Phenotypic plasticity in animals facilitates evolution by promoting adaptation to environmental fluctuations, colonization of novel habitats, species interaction, genetic diversification, and speciation [55, 56]. *C*. *elegans* exist primarily as dauer larva in natural populations [57], suggesting that they face intense selection pressure in environments with fluctuating and often limited resources. If the early dauer-inducing environment is predictive of future conditions, we could reasonably speculate that the cellular memory of the dauer-inducing trigger could result in a gene expression program that affords postdauer adults increased fitness in a future adverse environment. For example, starvation in early life may prime a postdauer animal to curtail fecundity in order to conserve resources and minimize the consequences of another impending famine on its progeny. In contrast, over-population could reliably predict increased opportunities of outcrossing in PD_Phe_ progeny and result in beneficial increases in the expression of reproduction-associated genes and higher brood size [58]. Thus, the phenotypic variation resulting from the pheromone and starvation programs in *C*. *elegans* may be the product of an evolutionary strategy to increase future fitness in response to past environmental challenges. Future studies will be necessary to determine whether the environmental effects on PD_Phe_ and PD_Stv_ parents are inherited and translated into heightened fitness in subsequent generations.

### Distinct life histories: Pheromone- and starvation-induced programs

Our results support a model where at least two distinct life history programs, the pheromone and starvation programs (Fig 7A), are orchestrated in postdauers through the exchange of signals between the germ line and the soma (Fig 7B). An intriguing question is what the candidate somatic and germline signals regulating the PD/CON programs might be. In the pheromone program, the requirement for SID-1 strongly implicates dsRNAs as mediators of gene expression changes in the soma and the germ line. Endogenous dsRNAs could potentially be processed into, or stimulate the production of, siRNAs that associate with CSR-1 in specific tissue types, analogous to how exogenous dsRNA results in target specific silencing by Argonautes such as NRDE-3 (WBGene00019862) [59]. Since one of the proposed functions of CSR-1 is the establishment and maintenance of euchromatic chromatin states associated with target genes, and we have shown that CSR-1 targets exhibiting similar gene expression patterns cluster at the chromosomal level (Fig 5D), it is reasonable to propose that CSR-1 would also modify chromatin states due to pheromone and starvation in a tissue-specific manner. Indeed, we previously showed that changes in the levels of two histone modifications associated with euchromatin in PD_Phe_/CON_Phe_ are dependent on CSR-1 [11]. These altered chromatin states could potentially spread along chromosomal regions including non-CSR-1 target genes, resulting chromosomal-biased changes in gene expression genome-wide (Fig 5).

In contrast, the starvation program is independent of SID-1 but dependent on the germ line (Fig 7B). One candidate mechanism for regulating the starvation-induced cellular memory is endocrine signaling, which plays a key role in lifespan and stress response in worms, flies, and mammals [60]. Effectors of endocrine signaling in *C*. *elegans* include the conserved FOXO transcription factor DAF-16 (WBGene00000912) and the nuclear hormone receptor DAF-12 (WBGene00000908), both of which function downstream of the insulin/IGF-1 signaling (IIS) pathway [61]. In *C*. *elegans*, *Drosophila*, and mice, removal or alteration of the germ line results in somatic aging phenotypes [60]. Nematodes with defective gonads exhibit extended lifespans that are dependent on DAF-16 activity in the intestine, as well as DAF-12 activity in the somatic gonad [60, 62], suggesting that the regulation of a somatic phenotype is mediated by germline signaling. Recent reports provide further evidence of such crosstalk: the chemotaxis response to the odorant diacetyl in *C*. *elegans* is dependent on DAF-16 and germline proliferation [63]; cold tolerance in worms is dependent on a feedback mechanism involving IIS signaling, temperature-sensing neurons, the intestine, and sperm cells [64] while a thermosensory neuronal circuit promotes longevity at warm temperatures by engaging endocrine signaling [65]; and the transgenerational lifespan extension in a mutant strain with defective chromatin remodeling is dependent on a germ line to soma signaling mechanism modulated by DAF-12 [66]. Therefore, endocrine signaling is an attractive contender with which distinct life history trajectories program a postdauer animal in response to early life starvation.

### Life history and the evolution of genome organization

Physical co-localization of genes with correlated expression patterns is widespread in eukaryotes [67] and is pronounced amongst genes expressed in the testis [68] and those encoding protein components of sperm [69]. Consistent with the efficient co-regulation of neighboring genes, we observed an excess of clustered genes in the central portions of chromosomes I, II, III, V, and X that exhibited WT_Phe_ up::WT_Stv_ down and WT_Phe_ down::WT_Stv_ up trends in gene expression changes (Fig 5A and 5D). Given the breadth of syntenic conservation between *C*. *elegans* and C. *briggsae* [70], it would appear likely that this genomic architecture arose in a common ancestor and may have contributed to reproductive fitness in what was likely to be an out-crossing species. Comparative genomic analyses will thus be essential in reconstructing the evolutionary history of PD reproductive investments using *C*. *briggsae*, which also shares a conserved CSR-1 RNAi pathway [38]. However, it is also noteworthy that the non-random organization of differentially expressed genes identified in this study overlaps substantively with regions of the *C*. *elegans* genome that experience limited recombination and large-scale selective sweeps [37]. Population genetic models predict that recurrent selective sweeps in linked regions would favor the establishment of adaptive “supergenes” over biologically realistic time scales [71, 72]; thus, we speculate that this process may have contributed to the expansion of regions harboring genes with coherent reproductive functions during *C*. *elegans* evolution [37, 73, 74].

## Materials and methods

### *C*. *elegans* strains and husbandry

The nematode strains are Bristol wild type strain N2, WM193 *csr-1*(*tm892)* IV; *neIs20* [*pie-1*::*3xFLAG*::*csr-1* + *unc-119*(+)], SS104 *glp-4(bn2)* I, HC196 *sid-1(qt9)* V, and BS1080 *ozIs5* [*gld-1*::*gfp/flag*, pMMO16 (*unc-119(+)*)] I. Worms were maintained using standard methods on Nematode Growth Medium (NGM) plates seeded with *Escherichia coli* OP50 at 20°C or at 15°C (for SS104 *glp-4(bn2)* I) [75].

To collect PD_Phe_, we used an egg white plate procedure described previously [76]. To obtain PD_Stv_, well-fed worms were transferred to seeded NGM plates and monitored until the *E*. *coli* OP50 food was depleted and the plates were populated with dauers. See Supplemental Experimental Procedures for details.

### Brood assays

Ten L4 larvae were singled onto seeded NGM plates and transferred daily onto fresh plates until egg laying ceased. Only surviving progeny were counted. At least three independent biological replicates were conducted. Statistical significance was determined using GraphPad Prism v.7.

### RNA extraction, RNA-seq library preparation, and data analyses

Total RNA extraction was done using TRIzol Reagent (Life Technologies). Two biological independent RNA-Seq libraries for a strain and a condition were prepared using the NEBNext mRNA Library Prep Master Mix Set for Illumina (NEB). Data analysis was conducted on the CLC Genomics Workbench v.8.5 (Qiagen) with differential expression calculated using EdgeR [77]. Detailed procedures are described in Supplemental Experimental Procedures.

### Gene ontology term analyses

GO terms were analyzed using DAVID (Database for Annotation, Visualization and Integrated Discovery) v.6.7 [78].

### Monte carlo simulation

The expected frequency of seesaw genes was investigated using a randomized approach whereby sets of genes, equal in number to the observed sets of differentially expressed genes under each condition, were selected from the whole genome set, without replacement, and their overlap assessed. The expected number of overlapping “seesaw” genes and the significance of the observed numbers were directly determined from the simulated distributions, based on 10,000 simulations.

### Quantitative reverse transcription PCR

qRT-PCR was done using samples collected from three independent biological replicates. S8 Table lists the primer sequences. Statistical analysis was done using GraphPad Prism v.7. Detailed procedures can be found in the Supplemental Experimental Procedures.

### Genomic clustering of CSR-1 targets and direction of change

The Global Landscape Clustering (GLC) algorithm (Borziak et al., manuscript in preparation) was used to identify maximal sets co-localized genes that share a specific attribute, such as being CSR-1 targets. See Supplemental Experimental Procedures for a detailed description. Significance of clustering was directly determined from the simulated distributions, based on 10,000 simulations.

### Sliding window analysis

Seesaw gene enrichment sliding window analyses were conducted on the EdgeR generated fold changes using gene position information based on the WormBase (WS235) annotations, using only coding transcripts. Sliding windows of 2.5 megabase pairs with 500 kilobase pair intervals were used. Seesaw genes were defined as those showing the inverse directional change of at least 2-fold, regardless of significance. Enrichment was calculated against the genome-wide average.

### Genome-wide gene expression graphs

Genome-wide gene expression graphs were generated using the log_2_ transformed EdgeR fold change values. Significance of correlation between experiments was calculated based on the Pearson's correlation between samples, where degrees of freedom equals (the number of genes in the plot– 1). Enrichment of directional change across chromosomes was calculated using χ^2^ test with Yates' correction against the remaining chromosomes.

### Germ line imaging and measurements

Detailed collection methods for BS1080 PD and CON larva in Phe and Stv conditions are described in S1 Text. For the Phe conditions, control and dauer larvae were collected using water or crude pheromone, respectively, on dauer formation plates as previously described [79; 80]. Images of larva exhibiting vulva morphology characteristic of the L3, L4.1, and L4.4 larval stages were analyzed for GLD-1::GFP expression and gonad area using ImageJ software (NIH). These same worms were next used for germ cell row counts using a standard whole worm DAPI staining protocol [81]. The size of mitotic zone, transition zone, pachytene zone was determined based on the germ cell nuclear morphology [82]. Statistical significance between CON and PD samples was determined using Student’s *t*-test.

### Accession numbers

The accession number for the high-throughput sequencing data reported in this study is GSE92954.

## Supporting information

S1 Fig Correlation plots for RNA-seq libraries. (A-H) Correlation plots for two independent biological replicates for (A) WT CON_Phe_, (B) WT PD_Phe_, (C) WT CON_Stv_, (D) WT PD_Stv_, (E) *csr-1* CON_Phe_, (F) *csr-1* PD_Phe_, (G) *csr-1* CON_Stv_, and (H) *csr-1* PD_Stv_ are shown. Pearson correlation coefficients (r) are indicated.
(TIF)

S2 Fig PCA for RNA-Seq library replicates. PCA was based on the total exon reads per gene, across 21666 genes. Significance of PC loadings per sample was assessed using the FactorMineR dimdesc function [86]. Significance of PCs was assessed using both the Kaiser criterion and the broken stick model [87]. For both the A) wild type and B) *csr-1* hypomorph PCA, only PC1 was significant using either test.
(TIF)

S3 Fig Comparison of differentially expressed WT_Phe_, WT_Stv_, *csr-1*_Phe_, *csr-1*_Stv_ seesaw genes and a germline-enriched data set. (A, B) Venn diagrams depicting the overlap between WT_Phe_ and WT_Stv_ DE genes. (C, D) Venn diagrams of the distribution of a germline-enriched gene set [19] with (C) WT_Phe_ down::WT_Stv_ up and (D) WT_Phe_ up::WT_Stv_ down seesaw genes. (E, F) Venn diagrams depicting the overlap between *csr-1*_Phe_ and *csr-1*_Stv_ DE genes.
(TIF)

S4 Fig qRT-PCR validation of RNA-seq results in wild-type populations. (A-D) qRT-PCR validation of (A) soma-enriched, CSR-1-targeted genes found not to be differentially expressed in WT_Phe_ and WT_Stv_, (B) soma-enriched, non-CSR-1-targeted seesaw genes, (C) germline-enriched, CSR-1-targeted seesaw genes, and (D) germline-enriched, non-CSR-1-targeted genes in wild-type animals. Measurements were performed in triplicates using three biologically independent samples. Error bars represent S.E.M. * *p* < 0.05, ** *p* < 0.01, *** *p* < 0.001, **** *p* < 0.0001; Student’s *t*-test comparison of Phe (PD_Phe_/CON_Phe_) and Stv (PD_Stv_/CON_Stv_). (E) Summary of the genes used for RNA-Seq validation.
(TIF)

S5 Fig qRT-PCR validation of RNA-seq results in the *csr-1* hypormorph. (A-C) qRT-PCR validation of (A) soma-enriched, CSR-1-targeted genes, (B) soma-enriched, non-CSR-1-targeted seesaw genes, and (C) germline-enriched, CSR-1-targeted seesaw genes in the *csr-1* hypomorph strain. Measurements were performed in triplicates using three biologically independent samples. Error bars represent S.E.M. ** *p* < 0.01; **** *p* < 0.0001; Student’s *t*-test comparison of Phe (PD_Phe_/CON_Phe_) and Stv (PD_Stv_/CON_Stv_).
(TIF)

S6 Fig qRT-PCR gene expression profiles in *glp-4(bn2)* animals. (A-C) qRT-PCR measurement of mRNA levels for (A) soma-enriched, CSR-1-targeted genes, (B) soma-enriched, non-CSR-1-targeted seesaw genes, and (C) germline-enriched, CSR-1-targeted genes in *glp-4(bn2)*. Measurements were performed in triplicates using three biologically independent samples. Error bars represent S.E.M. * *p* < 0.05; Student’s *t*-test comparison of Phe (PD_Phe_/CON_Phe_) and Stv (PD_Stv_/CON_Stv_).
(TIF)

S7 Fig qRT-PCR gene expression profiles in *sid-1(qt9)*. (A-C) qRT-PCR measurement of mRNA levels for (A) soma-enriched, CSR-1-targeted genes, (B) soma-enriched, non-CSR-targeted seesaw genes, and (C) germline-enriched, CSR-1-targeted genes in *sid-1(qt9)*. Measurements were performed in triplicates using three biologically independent samples. Error bars represent S.E.M. * *p* < 0.05, ** *p* < 0.01, *** *p* < 0.001, **** *p* < 0.0001; Student’s *t*-test comparison of Phe (PD_Phe_/CON_Phe_) and Stv (PD_Stv_/CON_Stv_).
(TIF)

S8 Fig GLD-1::GFP fluorescence and gonad arm area in larval stages. (A) Representative images of one gonad arm expressing GLD-1::GFP of CON and PD larva that exhibited larval L3 stage vulva morphology in Phe or Stv conditions. The dotted line indicates the outline of the germ line; arrowheads indicates examples of GLD-1::GFP. DTC, distal tip cell. (B, C) Corrected total cell fluorescence (CTCF) measurements and area of gonad arms in L3, L4.1, and L4.4 larva for (B) Phe and (C) Stv conditions. Line indicates the median of measurements within a sample. N indicates number of animals measured over 3 biologically independent trials. * *p* < 0.05, ** *p* < 0.01, *** *p* < 0.001, **** *p* < 0.0001; Student’s *t*-test.
(TIF)

S9 Fig Representative images of DAPI-stained gonad arms in Phe conditions. (A) Representative gonad arm images of live CON_Phe_ and PD_Phe_ animals at L4.1 and L4.4 stages with their corresponding vulva morphology in the insert. (B) Images of DAPI-stained gonad arms of CON_Phe_ and PD_Phe_ worms at the L3, L4.1 and L4.4 stages. The worms depicted in the L4.1 and L4.4 DAPI-stained images are from the same trial as the live worms shown in (A). L3 and L4.1 DAPI images were taken at 630X magnification, and L4.4 DAPI images were taken at 400X. The dotted line indicates the germ line; asterisk indicates the distal tip cell; MZ, mitotic zone; TZ, transition zone; PZ, pachytene zone; SC, spermathecal cell (part of the somatic gonad).
(TIF)

S10 Fig Representative images of DAPI-stained gonad arms in Stv conditions. (A) Representative gonad arm images of live CON_Stv_ and PD_Stv_ animals at L4.1 and L4.4 stages with their corresponding vulva morphology in the insert. (B) Images of DAPI-stained gonad arms of CON_Stv_ and PD_Stv_ worms at the L3, L4.1 and L4.4 stages. The worms depicted in the L4.1 and L4.4 DAPI-stained images are from the same trial as the live worms shown above in (A). L3 and L4.1 DAPI images were taken at 630X magnification, and L4.4 DAPI images were taken at 400X. The dotted line indicates the germ line; asterisk indicates the distal tip cell; MZ, mitotic zone; TZ, transition zone; PZ, pachytene zone; SC, spermathecal cell (part of the somatic gonad).
(TIF)

S1 Text Supplemental materials and methods. (DOCX)

S1 Table RNA-seq DE and genes overlapping with previous microarray study. (XLSX)

S2 Table List of seesaw genes. (XLSX)

S3 Table Overlap of germline-enriched data set with WT_Phe_ and WT_Stv_ seesaw genes. (XLSX)

S4 Table Curation of WT_Phe_ Up::WT_Stv_ down seesaw genes. (XLSX)

S5 Table Curation of WT_Phe_ Down::WT_Stv_ up seesaw genes. (XLSX)

S6 Table CSR-1 clustering genes and clustering of CSR-1 targets per chromosome. (XLSX)

S7 Table GO term analysis of sperm mRNAome and proteome genes associated with seesaw genes. (XLSX)

S8 Table Primers used for qRT-PCR. (XLSX)

S9 Table qRT-PCR data for Figs 3, 4, S3, S4, S5 and S6. (XLSX)

S10 Table Raw data for brood size assays in Fig 6. (XLSX)

S11 Table Raw data for GLD-1::GFP fluorescence and gonad arm area. (XLSX)

S12 Table Raw data for germ cell row counts. (XLSX)

## References

[1] Richards EJ. Inherited epigenetic variation—revisiting soft inheritance. Nat Rev Genet. 2006;7(5):395–401. doi: 10.1038/nrg1834 . 16534512 10.1038/nrg1834

[2] Kaati G, Bygren LO, Pembrey M, Sjostrom M. Transgenerational response to nutrition, early life circumstances and longevity. Eur J Hum Genet. 2007;15(7):784–90. doi: 10.1038/sj.ejhg.5201832 17457370 10.1038/sj.ejhg.5201832

[3] Rechavi O, Houri-Ze'evi L, Anava S, Goh WS, Kerk SY, Hannon GJ, et al Starvation-induced transgenerational inheritance of small RNAs in C. elegans. Cell. 2014;158(2):277–87. doi: 10.1016/j.cell.2014.06.020 ; PubMed Central PMCID: PMCPMC4377509. 25018105 10.1016/j.cell.2014.06.020 PMC4377509

[4] Xia B, de Belle JS. Transgenerational programming of longevity and reproduction by post-eclosion dietary manipulation in Drosophila. Aging (Albany NY). 2016;8(5):1115–34. doi: 10.18632/aging.100932 ; PubMed Central PMCID: PMCPMC4931857. 27025190 10.18632/aging.100932 PMC4931857

[5] Skinner MK. Environmental Epigenetics and a Unified Theory of the Molecular Aspects of Evolution: A Neo-Lamarckian Concept that Facilitates Neo-Darwinian Evolution. Genome Biol Evol. 2015;7(5):1296–302. doi: 10.1093/gbe/evv073 ; PubMed Central PMCID: PMCPMC4453068. 25917417 10.1093/gbe/evv073 PMC4453068

[6] Verhoeven KJ, vonHoldt BM, Sork VL. Epigenetics in ecology and evolution: what we know and what we need to know. Mol Ecol. 2016;25(8):1631–8. doi: 10.1111/mec.13617 . 26994410 10.1111/mec.13617

[7] Sulston JE, Horvitz HR. Post-embryonic cell lineages of the nematode, Caenorhabditis elegans. Dev Biol. 1977;56(1):110–56. . 838129 10.1016/0012-1606(77)90158-0

[8] Cassada RC, Russell RL. The dauerlarva, a post-embryonic developmental variant of the nematode Caenorhabditis elegans. Dev Biol. 1975;46(2):326–42. . 1183723 10.1016/0012-1606(75)90109-8

[9] Frezal L, Felix MA. C. elegans outside the Petri dish. Elife. 2015;4 doi: 10.7554/eLife.05849 ; PubMed Central PMCID: PMCPMC4373675. 25822066 10.7554/eLife.05849 PMC4373675

[10] Hall SE, Beverly M, Russ C, Nusbaum C, Sengupta P. A cellular memory of developmental history generates phenotypic diversity in C. elegans. Curr Biol. 2010;20(2):149–55. doi: 10.1016/j.cub.2009.11.035 ; PubMed Central PMCID: PMCPMC2990539. 20079644 10.1016/j.cub.2009.11.035 PMC2990539

[11] Hall SE, Chirn GW, Lau NC, Sengupta P. RNAi pathways contribute to developmental history-dependent phenotypic plasticity in C. elegans. RNA. 2013;19(3):306–19. doi: 10.1261/rna.036418.112 ; PubMed Central PMCID: PMCPMC3677242. 23329696 10.1261/rna.036418.112 PMC3677242

[12] Sims JR, Ow MC, Nishiguchi MA, Kim K, Sengupta P, Hall SE. Developmental programming modulates olfactory behavior in C. elegans via endogenous RNAi pathways. Elife. 2016;5 doi: 10.7554/eLife.11642 ; PubMed Central PMCID: PMCPMC4924998. 27351255 10.7554/eLife.11642 PMC4924998

[13] Weismann A. The germ-plasm: a theory of heredity: Charles Scribner's Sons; 1893.

[14] Anava S, Posner R, Rechavi O. The soft genome. Worm. 2014;3(4):e989798 doi: 10.4161/21624054.2014.989798 ; PubMed Central PMCID: PMCPMC4588383. 26430554 10.4161/21624054.2014.989798 PMC4588383

[15] Devanapally S, Ravikumar S, Jose AM. Double-stranded RNA made in C. elegans neurons can enter the germline and cause transgenerational gene silencing. Proc Natl Acad Sci U S A. 2015;112(7):2133–8. doi: 10.1073/pnas.1423333112 ; PubMed Central PMCID: PMCPMC4343102. 25646479 10.1073/pnas.1423333112 PMC4343102

[16] Bharadwaj PS, Hall SE. Endogenous RNAi Pathways Are Required in Neurons for Dauer Formation in Caenorhabditis elegans. Genetics. 2017 doi: 10.1534/genetics.116.195438 . 28122825 10.1534/genetics.116.195438 PMC5378109

[17] Jungkamp AC, Stoeckius M, Mecenas D, Grun D, Mastrobuoni G, Kempa S, et al In vivo and transcriptome-wide identification of RNA binding protein target sites. Mol Cell. 2011;44(5):828–40. doi: 10.1016/j.molcel.2011.11.009 ; PubMed Central PMCID: PMCPMC3253457. 22152485 10.1016/j.molcel.2011.11.009 PMC3253457

[18] Beanan MJ, Strome S. Characterization of a germ-line proliferation mutation in C. elegans. Development. 1992;116(3):755–66. . 1289064 10.1242/dev.116.3.755

[19] Grun D, Kirchner M, Thierfelder N, Stoeckius M, Selbach M, Rajewsky N. Conservation of mRNA and protein expression during development of C. elegans. Cell Rep. 2014;6(3):565–77. doi: 10.1016/j.celrep.2014.01.001 . 24462290 10.1016/j.celrep.2014.01.001

[20] Howe KL, Bolt BJ, Cain S, Chan J, Chen WJ, Davis P, et al WormBase 2016: expanding to enable helminth genomic research. Nucleic Acids Res. 2016;44(D1):D774–80. doi: 10.1093/nar/gkv1217 ; PubMed Central PMCID: PMCPMC4702863. 26578572 10.1093/nar/gkv1217 PMC4702863

[21] Celniker SE, Dillon LA, Gerstein MB, Gunsalus KC, Henikoff S, Karpen GH, et al Unlocking the secrets of the genome. Nature. 2009;459(7249):927–30. doi: 10.1038/459927a ; PubMed Central PMCID: PMCPMC2843545. 19536255 10.1038/459927a PMC2843545

[22] Yates A, Akanni W, Amode MR, Barrell D, Billis K, Carvalho-Silva D, et al Ensembl 2016. Nucleic Acids Res. 2016;44(D1):D710–6. doi: 10.1093/nar/gkv1157 ; PubMed Central PMCID: PMCPMC4702834. 26687719 10.1093/nar/gkv1157 PMC4702834

[23] Fernandez AG, Gunsalus KC, Huang J, Chuang LS, Ying N, Liang HL, et al New genes with roles in the C. elegans embryo revealed using RNAi of ovary-enriched ORFeome clones. Genome Res. 2005;15(2):250–9. doi: 10.1101/gr.3194805 ; PubMed Central PMCID: PMCPMC546526. 15687288 10.1101/gr.3194805 PMC546526

[24] Wang X, Zhao Y, Wong K, Ehlers P, Kohara Y, Jones SJ, et al Identification of genes expressed in the hermaphrodite germline of C. elegans using SAGE. BMC Genomics. 2009;10:213 doi: 10.1186/1471-2164-10-213 ; PubMed Central PMCID: PMCPMC2686737. 19426519 10.1186/1471-2164-10-213 PMC2686737

[25] Spencer WC, Zeller G, Watson JD, Henz SR, Watkins KL, McWhirter RD, et al A spatial and temporal map of C. elegans gene expression. Genome Res. 2011;21(2):325–41. doi: 10.1101/gr.114595.110 ; PubMed Central PMCID: PMCPMC3032935. 21177967 10.1101/gr.114595.110 PMC3032935

[26] Zugasti O, Thakur N, Belougne J, Squiban B, Kurz CL, Soule J, et al A quantitative genome-wide RNAi screen in C. elegans for antifungal innate immunity genes. BMC Biol. 2016;14:35 doi: 10.1186/s12915-016-0256-3 ; PubMed Central PMCID: PMCPMC4850687. 27129311 10.1186/s12915-016-0256-3 PMC4850687

[27] Avgousti DC, Palani S, Sherman Y, Grishok A. CSR-1 RNAi pathway positively regulates histone expression in C. elegans. EMBO J. 2012;31(19):3821–32. doi: 10.1038/emboj.2012.216 ; PubMed Central PMCID: PMCPMC3463841. 22863779 10.1038/emboj.2012.216 PMC3463841

[28] Cecere G, Hoersch S, O'Keeffe S, Sachidanandam R, Grishok A. Global effects of the CSR-1 RNA interference pathway on the transcriptional landscape. Nat Struct Mol Biol. 2014;21(4):358–65. doi: 10.1038/nsmb.2801 ; PubMed Central PMCID: PMCPMC4068146. 24681887 10.1038/nsmb.2801 PMC4068146

[29] Claycomb JM, Batista PJ, Pang KM, Gu W, Vasale JJ, van Wolfswinkel JC, et al The Argonaute CSR-1 and its 22G-RNA cofactors are required for holocentric chromosome segregation. Cell. 2009;139(1):123–34. doi: 10.1016/j.cell.2009.09.014 ; PubMed Central PMCID: PMCPMC2766185. 19804758 10.1016/j.cell.2009.09.014 PMC2766185

[30] Conine CC, Moresco JJ, Gu W, Shirayama M, Conte D Jr., Yates JR 3rd, et al Argonautes promote male fertility and provide a paternal memory of germline gene expression in C. elegans. Cell. 2013;155(7):1532–44. doi: 10.1016/j.cell.2013.11.032 ; PubMed Central PMCID: PMCPMC3924572. 24360276 10.1016/j.cell.2013.11.032 PMC3924572

[31] Seth M, Shirayama M, Gu W, Ishidate T, Conte D Jr., Mello CC. The C. elegans CSR-1 argonaute pathway counteracts epigenetic silencing to promote germline gene expression. Dev Cell. 2013;27(6):656–63. doi: 10.1016/j.devcel.2013.11.014 ; PubMed Central PMCID: PMCPMC3954781. 24360782 10.1016/j.devcel.2013.11.014 PMC3954781

[32] Wedeles CJ, Wu MZ, Claycomb JM. Protection of germline gene expression by the C. elegans Argonaute CSR-1. Dev Cell. 2013;27(6):664–71. doi: 10.1016/j.devcel.2013.11.016 . 24360783 10.1016/j.devcel.2013.11.016

[33] Winston WM, Molodowitch C, Hunter CP. Systemic RNAi in C. elegans requires the putative transmembrane protein SID-1. Science. 2002;295(5564):2456–9. doi: 10.1126/science.1068836 . 11834782 10.1126/science.1068836

[34] Feinberg EH, Hunter CP. Transport of dsRNA into cells by the transmembrane protein SID-1. Science. 2003;301(5639):1545–7. doi: 10.1126/science.1087117 . 12970568 10.1126/science.1087117

[35] Ghazi A. Transcriptional networks that mediate signals from reproductive tissues to influence lifespan. Genesis. 2013;51(1):1–15. doi: 10.1002/dvg.22345 . 22945891 10.1002/dvg.22345

[36] Joh RI, Khanduja JS, Calvo IA, Mistry M, Palmieri CM, Savol AJ, et al Survival in Quiescence Requires the Euchromatic Deployment of Clr4/SUV39H by Argonaute-Associated Small RNAs. Mol Cell. 2016;64(6):1088–101. doi: 10.1016/j.molcel.2016.11.020 ; PubMed Central PMCID: PMCPMC5180613. 27984744 10.1016/j.molcel.2016.11.020 PMC5180613

[37] Andersen EC, Gerke JP, Shapiro JA, Crissman JR, Ghosh R, Bloom JS, et al Chromosome-scale selective sweeps shape Caenorhabditis elegans genomic diversity. Nat Genet. 2012;44(3):285–90. doi: 10.1038/ng.1050 ; PubMed Central PMCID: PMCPMC3365839. 22286215 10.1038/ng.1050 PMC3365839

[38] Tu S, Wu MZ, Wang J, Cutter AD, Weng Z, Claycomb JM. Comparative functional characterization of the CSR-1 22G-RNA pathway in Caenorhabditis nematodes. Nucleic Acids Res. 2015;43(1):208–24. doi: 10.1093/nar/gku1308 ; PubMed Central PMCID: PMCPMC4288196. 25510497 10.1093/nar/gku1308 PMC4288196

[39] Reinke V, Cutter AD. Germline expression influences operon organization in the Caenorhabditis elegans genome. Genetics. 2009; 181:1219–28. doi: 10.1534/genetics.108.099283 19204375 10.1534/genetics.108.099283 PMC2666493

[40] Allen MA, Hillier LW, Waterston RH, Blumenthal T. A global analysis of C. elegans trans-splicing. Genome Res. 2011; 21:255–64. doi: 10.1101/gr.113811.110 21177958 10.1101/gr.113811.110 PMC3032929

[41] Rastogi S, Borgo B, Pazdernik N, Fox P, Mardis ER, Kohara Y, Havranek J, Schedl T. Caenorhabditis elegans glp-4 Encodes a Valyl Aminoacyl tRNA Synthetase. G3 (Bethesda). 2015; 5:2719–28. doi: 10.1534/g3.115.021899 . 26464357 10.1534/g3.115.021899 PMC4683644

[42] Hodgkin J. Sexual dimorphism and sex determination Wood WB, editor. Cold Spring Harbor, New York: Cold Spring Harbor Laboratory Press; 1988.

[43] Kimble JE, White JG. On the control of germ cell development in Caenorhabditis elegans. Dev Biol. 1981 1 30;81(2):208–19. . 7202837 10.1016/0012-1606(81)90284-0

[44] Austin J, Kimble J. glp-1 is required in the germline for regulation of the decision between mitosis and meiosis in C. elegans. Cell. 1987 11 20;51(4):589–99. . 3677168 10.1016/0092-8674(87)90128-0

[45] Biedermann B, Wright J, Senften M, Kalchhauser I, Sarathy G, Lee MH, Ciosk R. Translational repression of cyclin E prevents precocious mitosis and embryonic gene activation during C. elegans meiosis. Dev Cell. 2009 9;17(3):355–64. doi: 10.1016/j.devcel.2009.08.003 . 19758560 10.1016/j.devcel.2009.08.003

[46] Fox PM, Vought VE, Hanazawa M, Lee MH, Maine EM, Schedl T. Cyclin E and CDK-2 regulate proliferative cell fate and cell cycle progression in the C. elegans germline.Development. 2011 6;138(11):2223–34. doi: 10.1242/dev.059535 . 21558371 10.1242/dev.059535 PMC3091494

[47] Seydoux G, Savage C, Greenwald I. Isolation and characterization of mutations causing abnormal eversion of the vulva in Caenorhabditis elegans. Dev Biol. 1993 6;157(2):423–36. . 8500652 10.1006/dbio.1993.1146

[48] Mok DZ, Sternberg PW, Inoue T. Morphologically defined sub-stages of C. elegans vulval development in the fourth larval stage. BMC Dev Biol. 2015; 15:26 PMCID: PMC4464634 doi: 10.1186/s12861-015-0076-7 26066484 10.1186/s12861-015-0076-7 PMC4464634

[49] Brenner JL, Schedl T. Germline Stem Cell Differentiation Entails Regional Control of Cell Fate Regulator GLD-1 in Caenorhabditis elegans. Genetics. 2016 3;202(3):1085–103. doi: 10.1534/genetics.115.185678 . 26757772 10.1534/genetics.115.185678 PMC4788111

[50] Hansen D, Wilson-Berry L, Dang T, Schedl T. Control of the proliferation versus meiotic development decision in the C. elegans germline through regulation of GLD-1 protein accumulation. Development. 2004 1;131(1):93–104. Epub 2003 Dec 3. doi: 10.1242/dev.00916 . 14660440 10.1242/dev.00916

[51] Seydoux G, Savage C, Greenwald I. Isolation and characterization of mutations causing abnormal eversion of the vulva in Caenorhabditis elegans. Dev Biol. 1993; 157:423–36. . 8500652 10.1006/dbio.1993.1146

[52] Ahringer J, Kimble J. Control of the sperm-oocyte switch in Caenorhabditis elegans hermaphrodites by the fem-3 3' untranslated region. Nature 1991; 349: 346–8. doi: 10.1038/349346a0 . 1702880 10.1038/349346a0

[53] Riddle DL, Blumenthal T, Meyer BJ, et al, editors. Somatic gonad. Cold Spring Harbor (NY): Cold Spring Harbor Laboratory Press; 1997.

[54] Ma X, Zhu Y, Li C, Xue P, Zhao Y, Chen S, et al Characterisation of Caenorhabditis elegans sperm transcriptome and proteome. BMC Genomics. 2014;15:168 doi: 10.1186/1471-2164-15-168 ; PubMed Central PMCID: PMCPMC4028957. 24581041 10.1186/1471-2164-15-168 PMC4028957

[55] Agrawal AA. Phenotypic plasticity in the interactions and evolution of species. Science. 2001;294(5541):321–6. doi: 10.1126/science.1060701 . 11598291 10.1126/science.1060701

[56] Moczek AP, Sultan S, Foster S, Ledon-Rettig C, Dworkin I, Nijhout HF, et al The role of developmental plasticity in evolutionary innovation. Proc Biol Sci. 2011;278(1719):2705–13. doi: 10.1098/rspb.2011.0971 ; PubMed Central PMCID: PMCPMC3145196. 21676977 10.1098/rspb.2011.0971 PMC3145196

[57] Braendle C, Milloz J, Felix MA. Mechanisms and evolution of environmental responses in Caenorhabditis elegans. Curr Top Dev Biol. 2008;80:171–207. doi: 10.1016/S0070-2153(07)80005-6 . 17950375 10.1016/S0070-2153(07)80005-6

[58] Morran LT, Parmenter MD, Phillips PC. Mutation load and rapid adaptation favour outcrossing over self-fertilization. Nature 2009; 462: 350–352. doi: 10.1038/nature08496 . 19847164 10.1038/nature08496 PMC4183137

[59] Gu SG, Pak J, Guang S, Maniar JM, Kennedy S, Fire A. Amplification of siRNA in Caenorhabditis elegans generates a transgenerational sequence-targeted histone H3 lysine 9 methylation footprint. Nat Genet. 2012; 44:157–164. doi: 10.1038/ng.1039 . 22231482 10.1038/ng.1039 PMC3848608

[60] Kenyon C. A pathway that links reproductive status to lifespan in Caenorhabditis elegans. Ann N Y Acad Sci. 2010;1204:156–62. doi: 10.1111/j.1749-6632.2010.05640.x . 20738286 10.1111/j.1749-6632.2010.05640.x

[61] Antebi A. Steroid regulation of C. elegans diapause, developmental timing, and longevity. Curr Top Dev Biol. 2013;105:181–212. doi: 10.1016/B978-0-12-396968-2.00007-5 . 23962843 10.1016/B978-0-12-396968-2.00007-5

[62] Libina N, Berman JR, Kenyon C. Tissue-specific activities of C. elegans DAF-16 in the regulation of lifespan. Cell. 2003;115(4):489–502. . 14622602 10.1016/s0092-8674(03)00889-4

[63] Fujiwara M, Aoyama I, Hino T, Teramoto T, Ishihara T. Gonadal Maturation Changes Chemotaxis Behavior and Neural Processing in the Olfactory Circuit of Caenorhabditis elegans. Curr Biol. 2016;26(12):1522–31. doi: 10.1016/j.cub.2016.04.058 . 27265391 10.1016/j.cub.2016.04.058

[64] Sonoda S, Ohta A, Maruo A, Ujisawa T, Kuhara A. Sperm Affects Head Sensory Neuron in Temperature Tolerance of Caenorhabditis elegans. Cell Rep. 2016;16(1):56–65. doi: 10.1016/j.celrep.2016.05.078 . 27320929 10.1016/j.celrep.2016.05.078

[65] Chen YC, Chen HJ, Tseng WC, Hsu JM, Huang TT, Chen CH, et al A C. elegans Thermosensory Circuit Regulates Longevity through crh-1/CREB-Dependent flp-6 Neuropeptide Signaling. Dev Cell. 2016;39(2):209–23. doi: 10.1016/j.devcel.2016.08.021 . 27720609 10.1016/j.devcel.2016.08.021

[66] Greer EL, Becker B, Latza C, Antebi A, Shi Y. Mutation of C. elegans demethylase spr-5 extends transgenerational longevity. Cell Res. 2016;26(2):229–38. doi: 10.1038/cr.2015.148 ; PubMed Central PMCID: PMCPMC4746603. 26691751 10.1038/cr.2015.148 PMC4746603

[67] Hurst LD, Pal C, Lercher MJ. The evolutionary dynamics of eukaryotic gene order. Nat Rev Genet. 2004;5(4):299–310. doi: 10.1038/nrg1319 . 15131653 10.1038/nrg1319

[68] Boutanaev AM, Kalmykova AI, Shevelyov YY, Nurminsky DI. Large clusters of co-expressed genes in the Drosophila genome. Nature. 2002;420(6916):666–9. doi: 10.1038/nature01216 . 12478293 10.1038/nature01216

[69] Dorus S, Busby SA, Gerike U, Shabanowitz J, Hunt DF, Karr TL. Genomic and functional evolution of the Drosophila melanogaster sperm proteome. Nat Genet. 2006;38(12):1440–5. doi: 10.1038/ng1915 . 17099714 10.1038/ng1915

[70] Vergara IA, Chen N. Large synteny blocks revealed between Caenorhabditis elegans and Caenorhabditis briggsae genomes using OrthoCluster. BMC Genomics. 2010 7 9;11:516 doi: 10.1186/1471-2164-11-516 20868500 10.1186/1471-2164-11-516 PMC2997010

[71] Yeaman S. Genomic rearrangements and the evolution of clusters of locally adaptive loci. Proc Natl Acad Sci U S A. 2013;110(19):E1743–51. doi: 10.1073/pnas.1219381110 ; PubMed Central PMCID: PMCPMC3651494. 23610436 10.1073/pnas.1219381110 PMC3651494

[72] Yeaman S, Aeschbacher S, Burger R. The evolution of genomic islands by increased establishment probability of linked alleles. Mol Ecol. 2016;25(11):2542–58. doi: 10.1111/mec.13611 . 27206531 10.1111/mec.13611

[73] Barnes TM, Kohara Y, Coulson A, Hekimi S. Meiotic recombination, noncoding DNA and genomic organization in Caenorhabditis elegans. Genetics. 1995;141(1):159–79. ; PubMed Central PMCID: PMCPMC1206715. 8536965 10.1093/genetics/141.1.159 PMC1206715

[74] Rockman MV, Kruglyak L. Recombinational landscape and population genomics of Caenorhabditis elegans. PLoS Genet. 2009;5(3):e1000419 doi: 10.1371/journal.pgen.1000419 ; PubMed Central PMCID: PMCPMC2652117. 19283065 10.1371/journal.pgen.1000419 PMC2652117

[75] Brenner S. The genetics of Caenorhabditis elegans. Genetics. 1974;77(1):71–94. ; PubMed Central PMCID: PMCPMC1213120. 4366476 10.1093/genetics/77.1.71 PMC1213120

[76] Ow MC, Hall SE. A Method for Obtaining Large Populations of Synchronized Caenorhabditis elegans Dauer Larvae. Methods Mol Biol. 2015;1327:209–19. doi: 10.1007/978-1-4939-2842-2_15 . 26423977 10.1007/978-1-4939-2842-2_15

[77] Robinson MD, McCarthy DJ, Smyth GK. edgeR: a Bioconductor package for differential expression analysis of digital gene expression data. Bioinformatics. 2010;26(1):139–40. doi: 10.1093/bioinformatics/btp616 ; PubMed Central PMCID: PMCPMC2796818. 19910308 10.1093/bioinformatics/btp616 PMC2796818

[78] Huang da W, Sherman BT, Lempicki RA. Systematic and integrative analysis of large gene lists using DAVID bioinformatics resources. Nat Protoc. 2009;4(1):44–57. doi: 10.1038/nprot.2008.211 . 19131956 10.1038/nprot.2008.211

[79] Neal SJ, Kim K, Sengupta P. Quantitative assessment of pheromone-induced dauer formation in Caenorhabditis elegans. Methods Mol Biol. 2013;1068:273–83. doi: 10.1007/978-1-62703-619-1_20 . 24014369 10.1007/978-1-62703-619-1_20

[80] Zhang X, Noguez JH, Zhou Y, Butcher RA. Analysis of ascarosides from *Caenorhabditis elegans* using mass spectrometry and NMR spectroscopy. Methods Mol Biol. 2013;1068:71–92. doi: 10.1007/978-1-62703-619-1_6 . 24014355 10.1007/978-1-62703-619-1_6 PMC3947767

[81] Qiao L, Lissemore JL, Shu P, Smardon A, Gelber MB, Maine EM. Enhancers of glp-1, a gene required for cell-signaling in Caenorhabditis elegans, define a set of genes required for germline development. Genetics. 1995;141:551–69. . 8647392 10.1093/genetics/141.2.551 PMC1206755

[82] Shakes DC, Wu JC, Sadler PL, Laprade K, Moore LL, Noritake A, Chu DS. Spermatogenesis-specific features of the meiotic program in Caenorhabditis elegans. PLoS Genet. 2009 8;5(8):e1000611 doi: 10.1371/journal.pgen.1000611 19696886 10.1371/journal.pgen.1000611 PMC2720455

[83] Page AP, Johnstone IL. The cuticle. WormBook. 2007:1–15. doi: 10.1895/wormbook.1.138.1 ; PubMed Central PMCID: PMCPMC4781593. 18050497 10.1895/wormbook.1.138.1 PMC4781593

[84] Budovskaya YV, Wu K, Southworth LK, Jiang M, Tedesco P, Johnson TE, et al An elt-3/elt-5/elt-6 GATA transcription circuit guides aging in C. elegans. Cell. 2008;134(2):291–303. doi: 10.1016/j.cell.2008.05.044 ; PubMed Central PMCID: PMCPMC4719053. 18662544 10.1016/j.cell.2008.05.044 PMC4719053

[85] Zhang Y, Zou X, Ding Y, Wang H, Wu X, Liang B. Comparative genomics and functional study of lipid metabolic genes in Caenorhabditis elegans. BMC Genomics. 2013;14:164 doi: 10.1186/1471-2164-14-164 ; PubMed Central PMCID: PMCPMC3602672. 23496871 10.1186/1471-2164-14-164 PMC3602672

[86] Husson F, Josse J, Le S, Mazet J. Package FactorMineR. doi: 10.1201/b10345-2

[87] Jackson DA. Stopping rules in principal components analysis: a comparison of heuristical and statistical approaches. Ecology. 1993; 74:2204–2214. doi: 10.2307/1939574

