# Supplementary figures and images for: Early experiences mediate distinct adult gene expression and reproductive programs in Caenorhabditis elegans
Source: PLoS Genet. 2018 Feb 15;14(2):e1007219. doi: 10.1371/journal.pgen.1007219 (PMC5831748; doi:10.1371/journal.pgen.1007219)

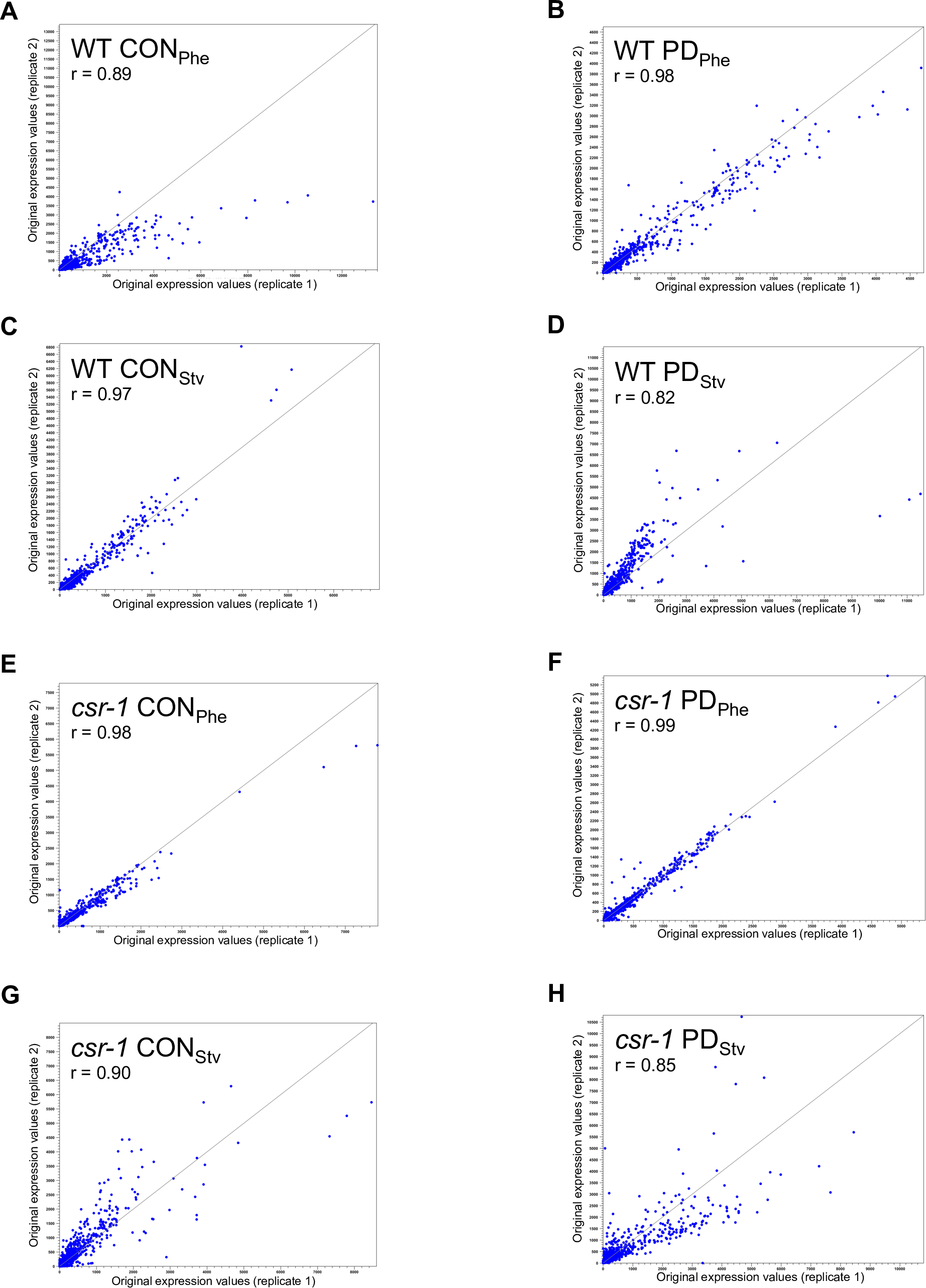

Supplement: S1 Fig — (A-H) Correlation plots for two independent biological replicates for (A) WT CONPhe, (B) WT PDPhe, (C) WT CONStv, (D) WT PDStv, (E) csr-1 CONPhe, (F) csr-1 PDPhe, (G) csr-1 CONStv, and (H) csr-1 PDStv are shown. Pearson correlation coefficients (r) are indicated. (TIF) [file pgen.1007219.s001.tif]

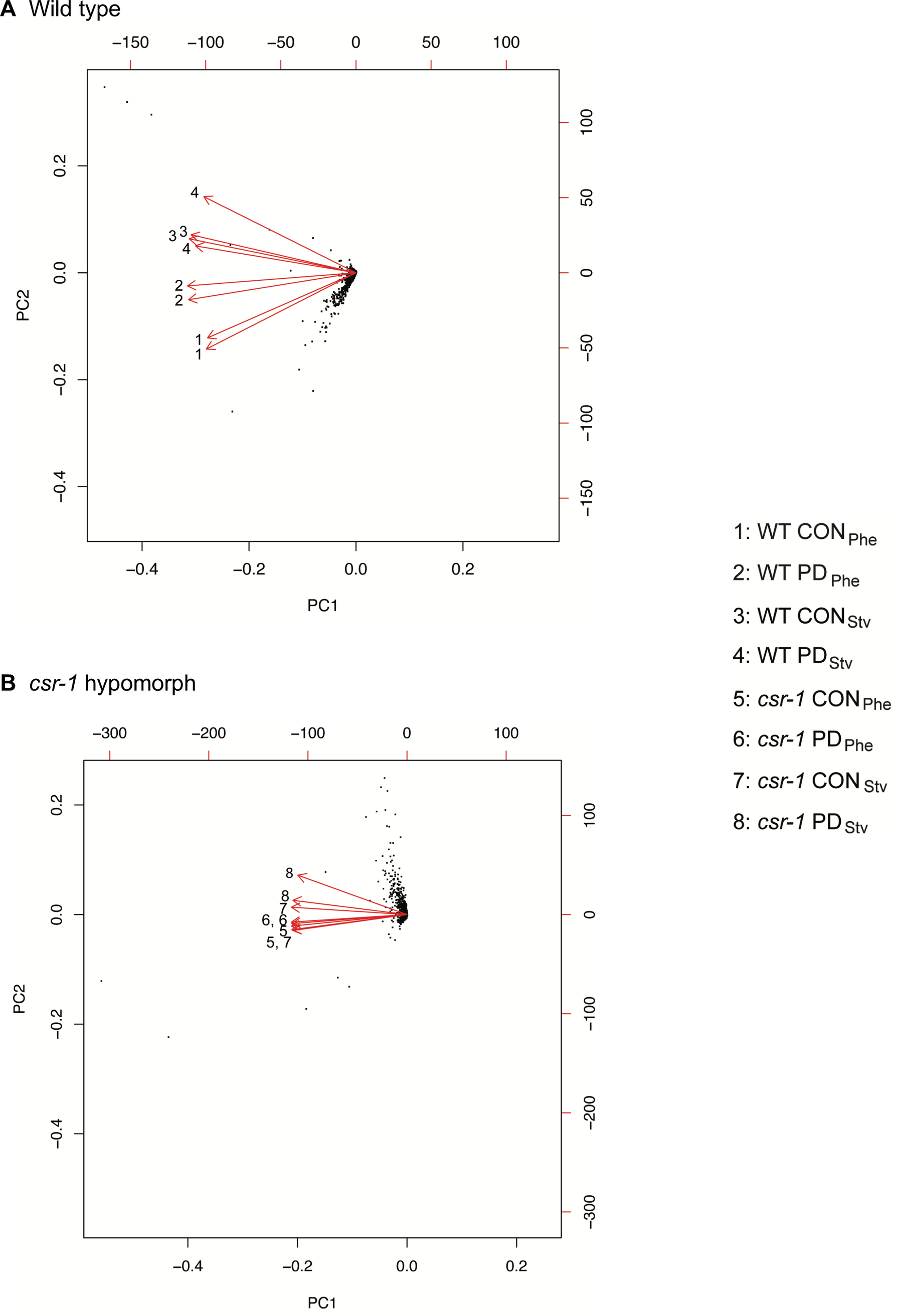

Supplement: S2 Fig — PCA was based on the total exon reads per gene, across 21666 genes. Significance of PC loadings per sample was assessed using the FactorMineR dimdesc function [86]. Significance of PCs was assessed using both the Kaiser criterion and the broken stick model [87]. For both the A) wild type and B) csr-1 hypomorph PCA, only PC1 was significant using either test. (TIF) [file pgen.1007219.s002.tif]

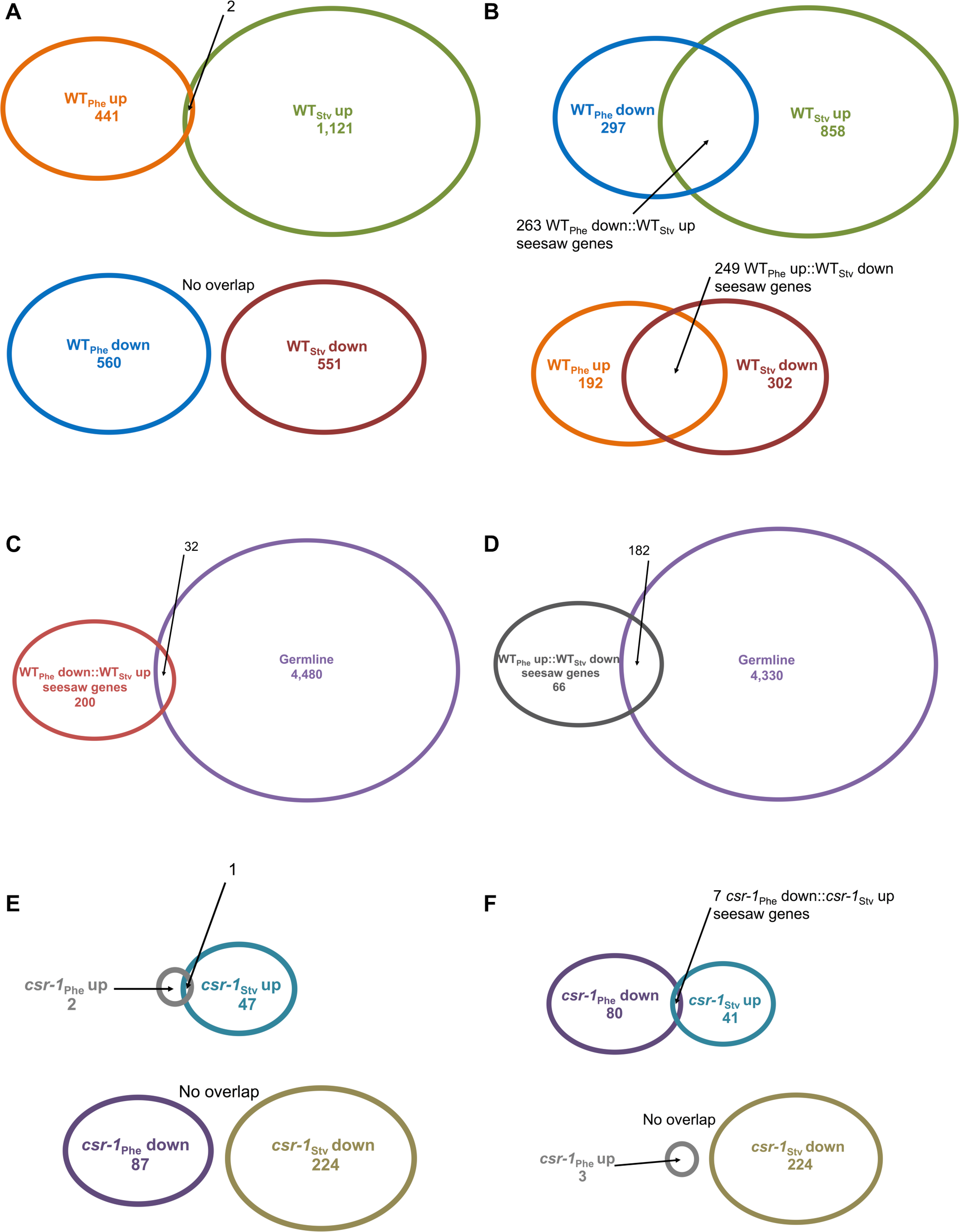

Supplement: S3 Fig — (A, B) Venn diagrams depicting the overlap between WTPhe and WTStv DE genes. (C, D) Venn diagrams of the distribution of a germline-enriched gene set [19] with (C) WTPhe down::WTStv up and (D) WTPhe up::WTStv down seesaw genes. (E, F) Venn diagrams depicting the overlap between csr-1Phe and csr-1Stv DE genes. (TIF) [file pgen.1007219.s003.tif]

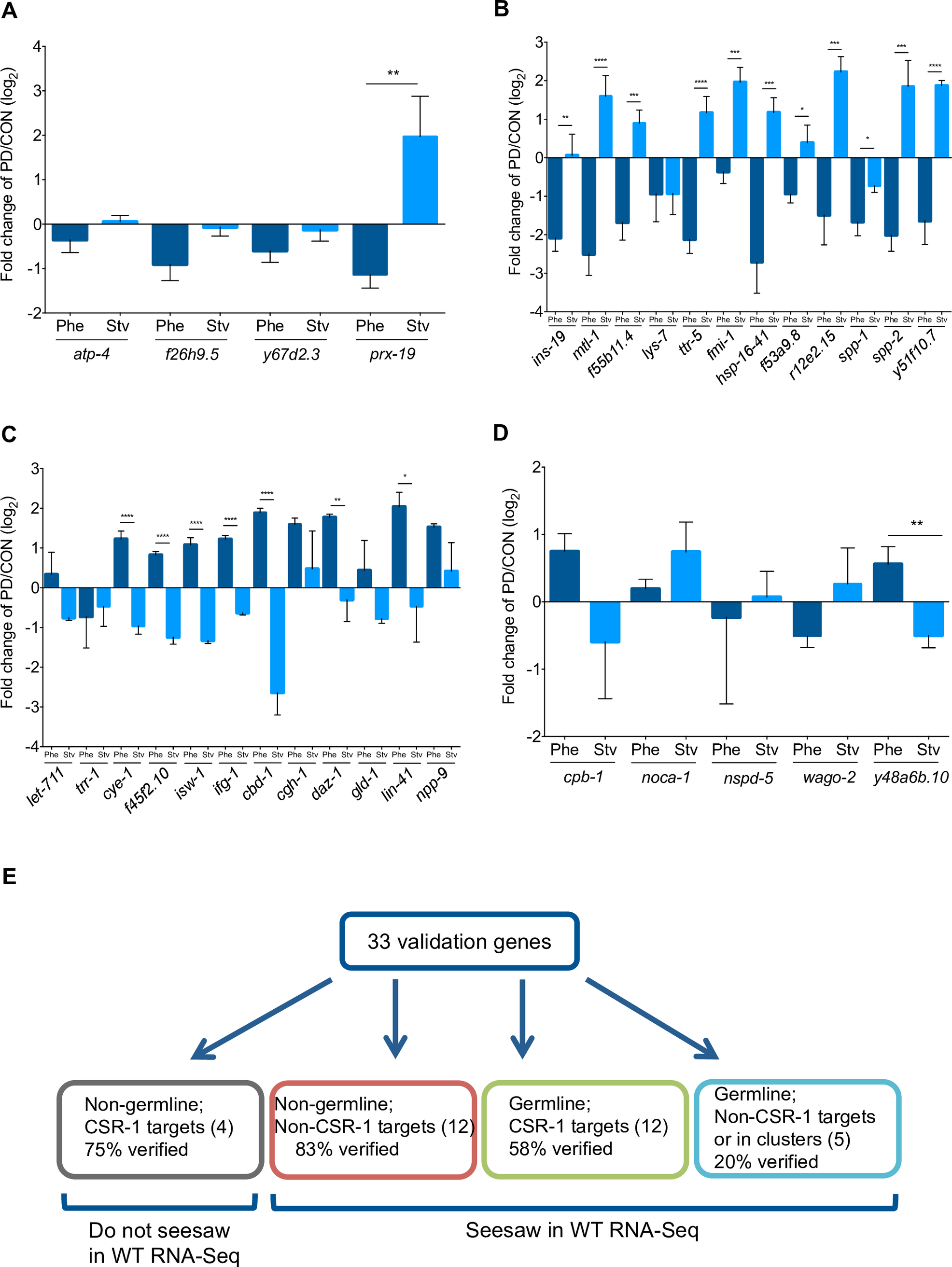

Supplement: S4 Fig — (A-D) qRT-PCR validation of (A) soma-enriched, CSR-1-targeted genes found not to be differentially expressed in WTPhe and WTStv, (B) soma-enriched, non-CSR-1-targeted seesaw genes, (C) germline-enriched, CSR-1-targeted seesaw genes, and (D) germline-enriched, non-CSR-1-targeted genes in wild-type animals. Measurements were performed in triplicates using three biologically independent samples. Error bars represent S.E.M. * p < 0.05, ** p < 0.01, *** p < 0.001, **** p < 0.0001; Student’s t-test comparison of Phe (PDPhe/CONPhe) and Stv (PDStv/CONStv). (E) Summary of the genes used for RNA-Seq validation. (TIF) [file pgen.1007219.s004.tif]

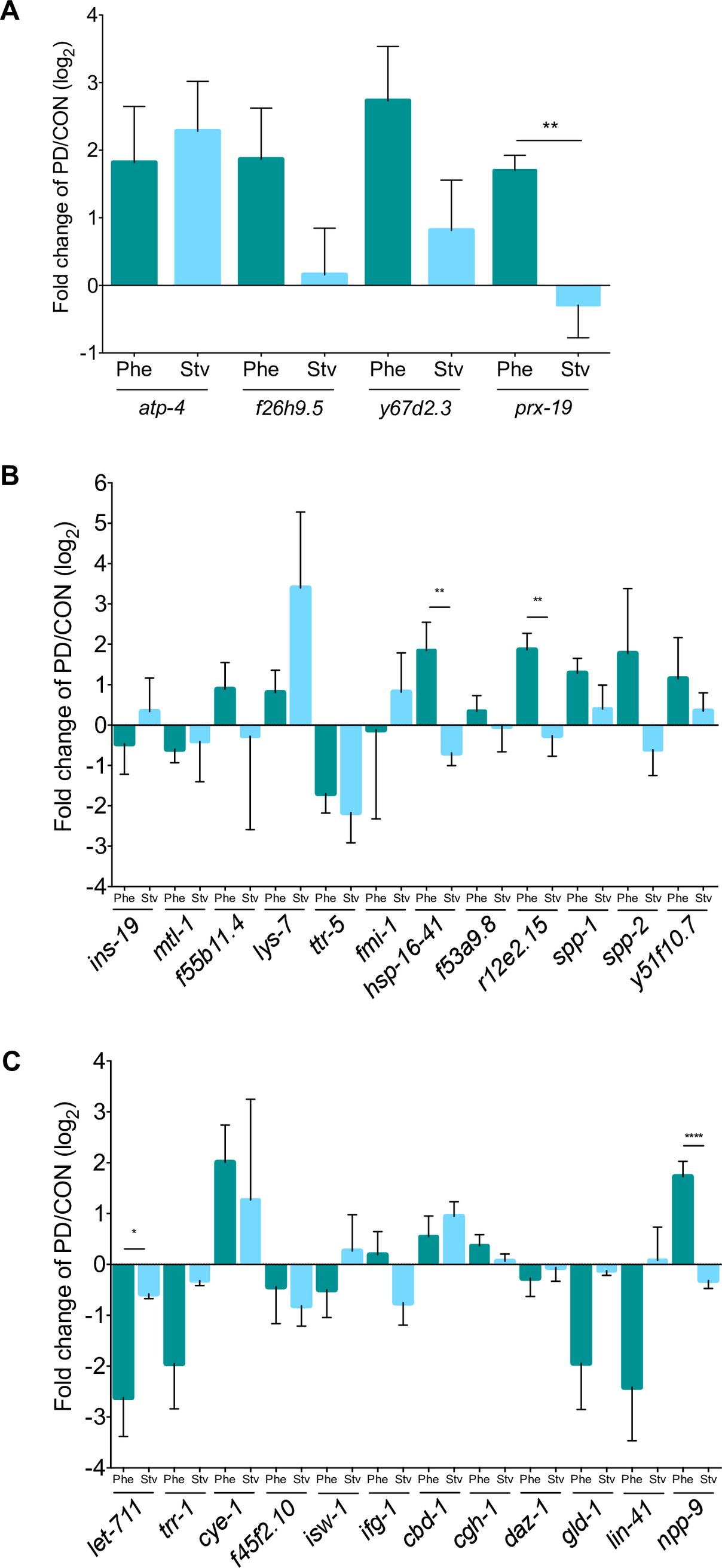

Supplement: S5 Fig — (A-C) qRT-PCR validation of (A) soma-enriched, CSR-1-targeted genes, (B) soma-enriched, non-CSR-1-targeted seesaw genes, and (C) germline-enriched, CSR-1-targeted seesaw genes in the csr-1 hypomorph strain. Measurements were performed in triplicates using three biologically independent samples. Error bars represent S.E.M. ** p < 0.01; **** p < 0.0001; Student’s t-test comparison of Phe (PDPhe/CONPhe) and Stv (PDStv/CONStv). (TIF) [file pgen.1007219.s005.tif]

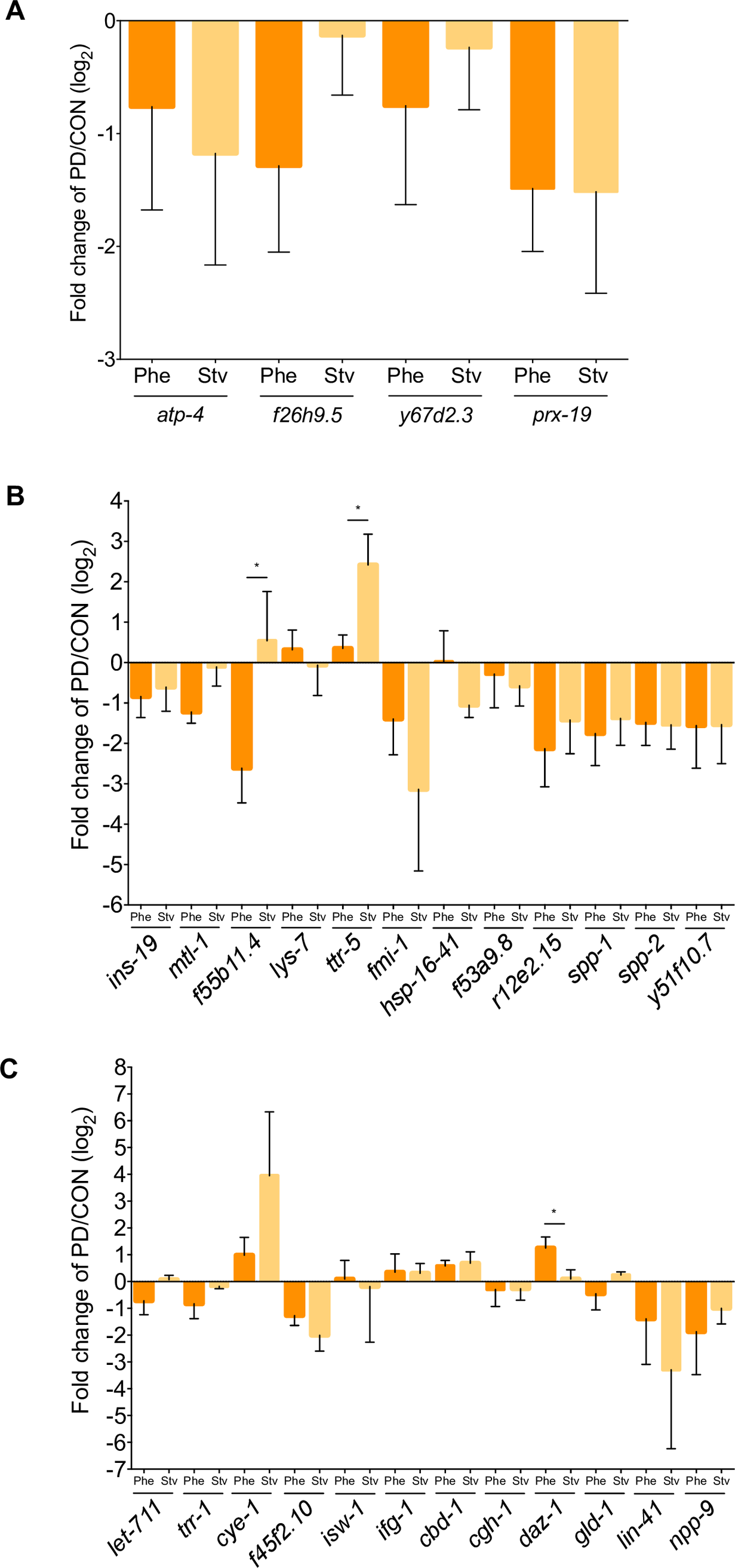

Supplement: S6 Fig — (A-C) qRT-PCR measurement of mRNA levels for (A) soma-enriched, CSR-1-targeted genes, (B) soma-enriched, non-CSR-1-targeted seesaw genes, and (C) germline-enriched, CSR-1-targeted genes in glp-4(bn2). Measurements were performed in triplicates using three biologically independent samples. Error bars represent S.E.M. * p < 0.05; Student’s t-test comparison of Phe (PDPhe/CONPhe) and Stv (PDStv/CONStv). (TIF) [file pgen.1007219.s006.tif]

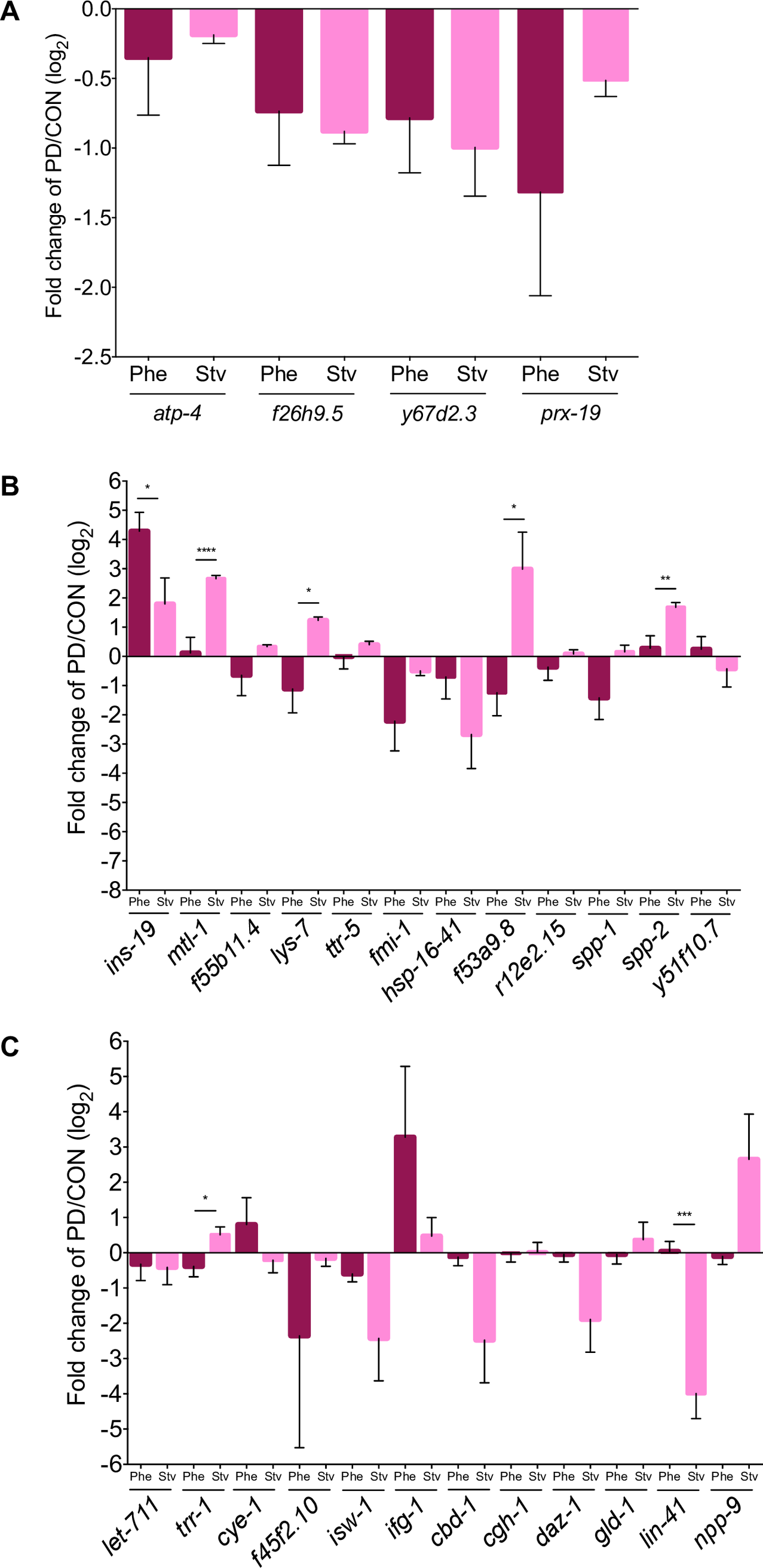

Supplement: S7 Fig — (A-C) qRT-PCR measurement of mRNA levels for (A) soma-enriched, CSR-1-targeted genes, (B) soma-enriched, non-CSR-targeted seesaw genes, and (C) germline-enriched, CSR-1-targeted genes in sid-1(qt9). Measurements were performed in triplicates using three biologically independent samples. Error bars represent S.E.M. * p < 0.05, ** p < 0.01, *** p < 0.001, **** p < 0.0001; Student’s t-test comparison of Phe (PDPhe/CONPhe) and Stv (PDStv/CONStv). (TIF) [file pgen.1007219.s007.tif]

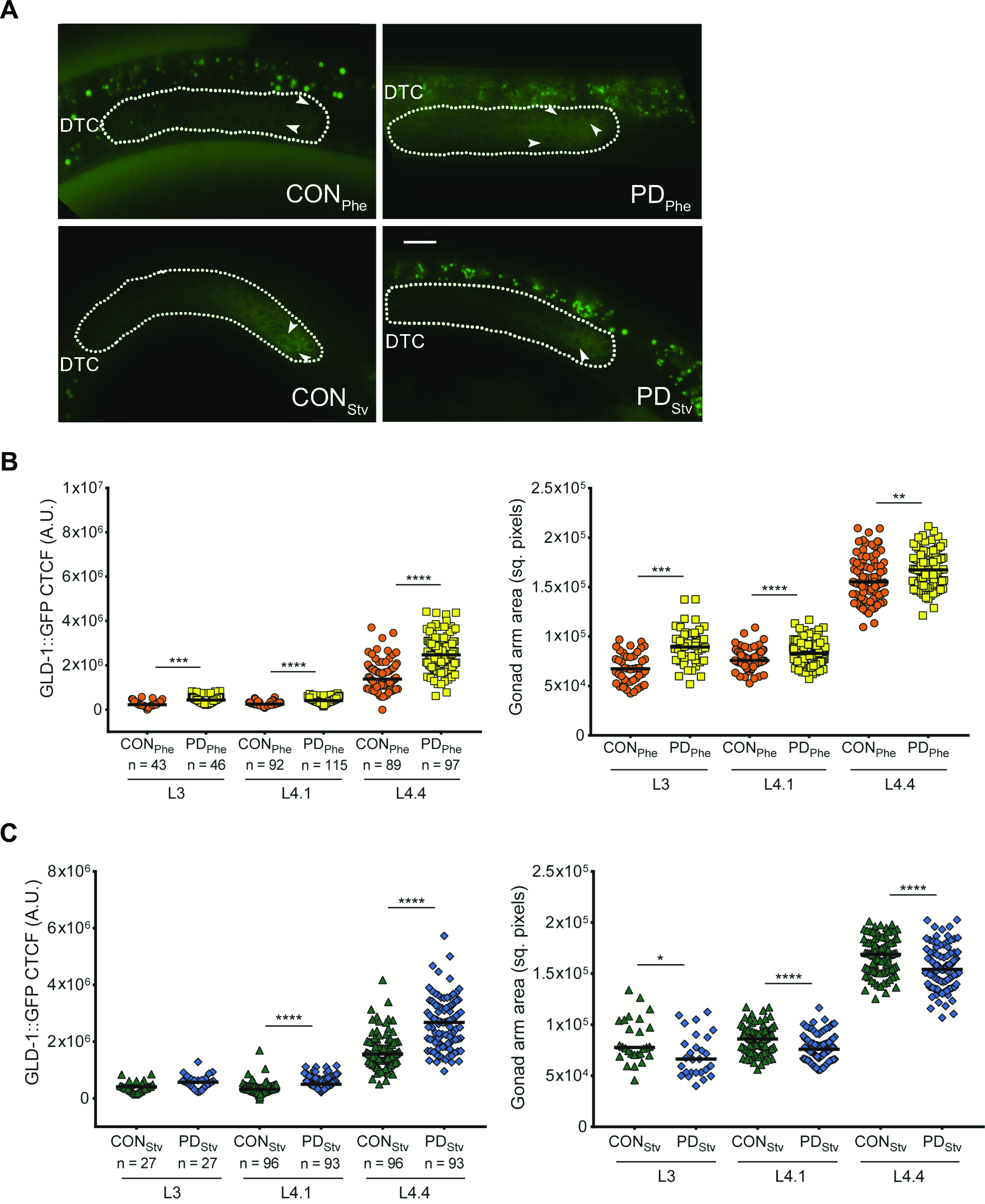

Supplement: S8 Fig — (A) Representative images of one gonad arm expressing GLD-1::GFP of CON and PD larva that exhibited larval L3 stage vulva morphology in Phe or Stv conditions. The dotted line indicates the outline of the germ line; arrowheads indicates examples of GLD-1::GFP. DTC, distal tip cell. (B, C) Corrected total cell fluorescence (CTCF) measurements and area of gonad arms in L3, L4.1, and L4.4 larva for (B) Phe and (C) Stv conditions. Line indicates the median of measurements within a sample. N indicates number of animals measured over 3 biologically independent trials. * p < 0.05, ** p < 0.01, *** p < 0.001, **** p < 0.0001; Student’s t-test. (TIF) [file pgen.1007219.s008.tif]

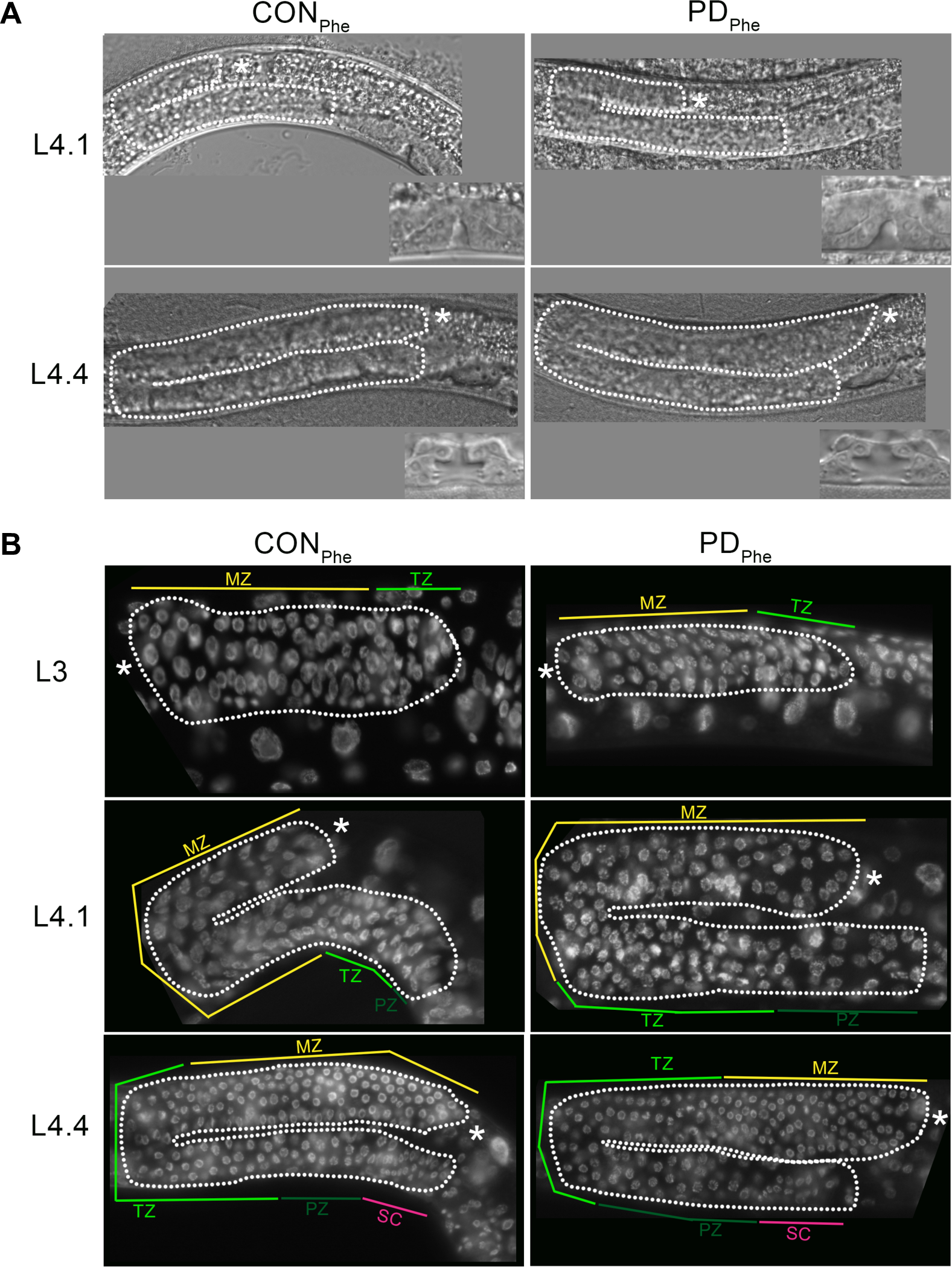

Supplement: S9 Fig — (A) Representative gonad arm images of live CONPhe and PDPhe animals at L4.1 and L4.4 stages with their corresponding vulva morphology in the insert. (B) Images of DAPI-stained gonad arms of CONPhe and PDPhe worms at the L3, L4.1 and L4.4 stages. The worms depicted in the L4.1 and L4.4 DAPI-stained images are from the same trial as the live worms shown in (A). L3 and L4.1 DAPI images were taken at 630X magnification, and L4.4 DAPI images were taken at 400X. The dotted line indicates the germ line; asterisk indicates the distal tip cell; MZ, mitotic zone; TZ, transition zone; PZ, pachytene zone; SC, spermathecal cell (part of the somatic gonad). (TIF) [file pgen.1007219.s009.tif]

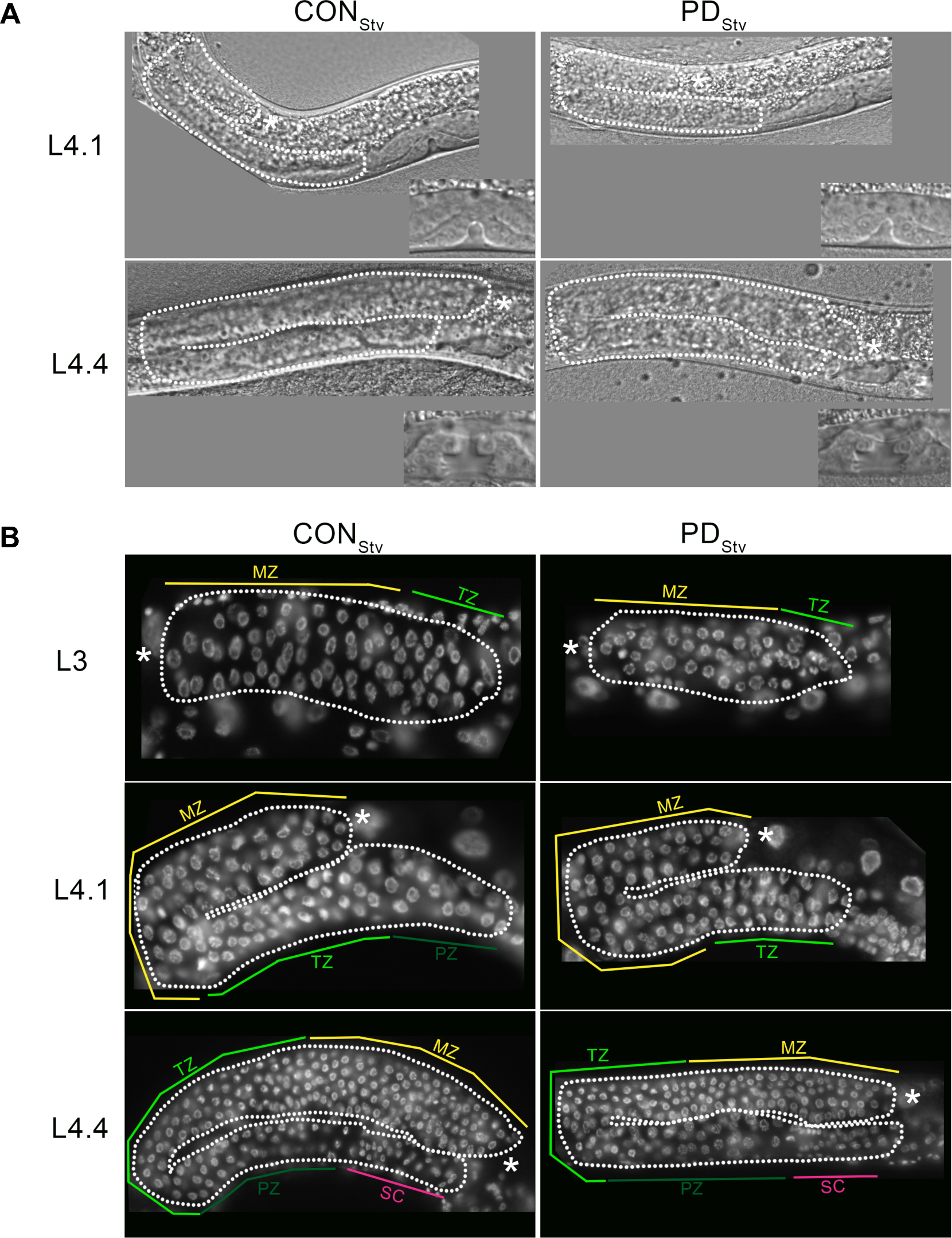

Supplement: S10 Fig — (A) Representative gonad arm images of live CONStv and PDStv animals at L4.1 and L4.4 stages with their corresponding vulva morphology in the insert. (B) Images of DAPI-stained gonad arms of CONStv and PDStv worms at the L3, L4.1 and L4.4 stages. The worms depicted in the L4.1 and L4.4 DAPI-stained images are from the same trial as the live worms shown above in (A). L3 and L4.1 DAPI images were taken at 630X magnification, and L4.4 DAPI images were taken at 400X. The dotted line indicates the germ line; asterisk indicates the distal tip cell; MZ, mitotic zone; TZ, transition zone; PZ, pachytene zone; SC, spermathecal cell (part of the somatic gonad). (TIF) [file pgen.1007219.s010.tif]
