# Supplementary material for: Early experiences mediate distinct adult gene expression and reproductive programs in Caenorhabditis elegans
Source: PLoS Genet. 2018 Feb 15;14(2):e1007219. doi: 10.1371/journal.pgen.1007219 (PMC5831748; doi:10.1371/journal.pgen.1007219)
Supplement: S1 Text — (DOCX) [file pgen.1007219.s011.docx]

**Early experiences mediate distinct adult gene expression and**

**reproductive programs in *Caenorhabditis elegans***

**Maria C. Ow, Kirill Borziak, Alexandra M. Nichitean, Steve Dorus, and Sarah E. Hall**

**Supplemental Materials and Methods**

**Preparation and collection of animal populations**

To induce dauers by crowding (high pheromone), we used an egg white plate procedure designed to yield a large number of staged postdauer adults [76]. A dense population of mixed stage worms was transferred to egg white plates and incubated for 3 days at its appropriate culturing temperature to induce dauer entry, followed by an overnight treatment with 1% SDS. Dauers were recovered and rescued by transferring them to seeded NGM plates. Two days following recovery from the dauer stage, one-day-old postdauer adults (PD_Phe_) were collected and flash frozen for downstream applications (see below). Control adults (CON_Phe_) used as comparison with PD_Phe_ adults were obtained by plating the embryos obtained by standard hypochlorite treatment [88] of well-fed gravid adults that did not experience the dauer stage onto seeded NGM plates containing egg white mixture on one side of the plate. After ~24 hours when the population was enriched for L2/L3 stage larva, control animals were recovered and transferred to seeded NGM plates. CON_Phe_ adults were collected as one-day old adults (24 hours after mid-L4 larval stage) and flash frozen. Although PD_Phe_ adults are chronologically 1 day older than CON_Phe_ adults due to 24 hours in dauer, these populations were collected at the same developmental stage.

To obtain starvation-induced postdauer adults, well-fed worms were transferred to seeded NGM plates and monitored until the *E. coli* OP50 food was recently exhausted and the plates were populated with dauers. This induction routinely took about one week. Worms were collected and dauers were selected for with 1% SDS treatment followed by rescue with feeding on seeded NGM plates. One-day-old young postdauer adults (PD_Stv_) were collected on the second day after feeding. CON_Stv_ adults were obtained by harvesting embryos from hypochlorite treated well-fed gravid adults that did not passage through the dauer stage and plated onto seeded NGM plates until the first day of adulthood (24 hours after mid-L4 larval stage). Similar to the Phe conditions, PD_Stv_ adults are chronologically 1 day older than CON_Stv_ adults; however, these populations were collected at the same developmental stage.

**RNA extraction, RNA-seq library preparation, sequencing, and data analyses**

Total RNA was extracted using TRIzol Reagent (Life Technologies). Four volumes of TRIzol reagent were added to a frozen worm pellet followed by vigorous vortexing for 20 minutes. Samples were centrifuged in a tabletop centrifuge at maximum speed, the cleared supernatant was transferred to a fresh tube, and the RNA precipitated with equal volume of isopropanol at -80^o^C for at least 30 minutes. The pellet was washed with cold 75% ethanol, dried, and resuspended in RNase-free water. The integrity of the RNA was determined by visualizing the ribosomal RNAs in an agarose gel.

Poly(A) RNA was selected from 20 μg of total RNA using the NEBNext Poly (A) mRNA Magnetic Isolation Module (NEB) as instructed by the manufacturer. Libraries were prepared with the NEBNext mRNA Library Prep Master Mix Set for Illumina (NEB). The mRNA was fragmented and purified using the RNeasy MinElute Cleanup Kit (Qiagen). Their size distribution, yield, and quality were determined using a BioAnalyzer RNA Pico chip (Agilent). A first strand cDNA was generated from the mRNA using random primers and ProtoScript II Reverse Transcriptase followed by a second strand cDNA synthesis step. The resulting double-stranded cDNA was purified using AMPure XP beads (Agilent), end-repaired, and dA-tailed. The cDNA library was purified, ligated with the NEBNext adaptor, and purified again. The adaptor-ligated DNA was sized selected with AMPure XP beads, PCR enriched using a high-fidelity DNA polymerase, and indexed using the NEBNext Multiplex Oligos for Illumina Index Primers Set 1 or 2 (NEB). The amplified library was purified and its quality assessed on a BioAnalyzer High Sensitivity DNA Chip (Agilent). To assess the quality of a library (enrichment for non-ribosomal RNA genes), an aliquot of a finished library was TOPO cloned (Life Technologies) and Sanger sequenced. The libraries were sequenced on an Illumina Hi-Seq 2000 using 50 base pair single reads. Analyses were done using the CLC Genomics Workbench v.8.5 (Qiagen) with *C. elegans* genome WS235 using the standard parameters of the Empirical analysis of DGE algorithm (EdgeR test) and a Benjamini-Hochberg FDR corrected *p*-value cutoff of < 0.05.

Analyses of RNA-Seq libraries yielded the following: 17,499 (67%) and 16,643 (64%) transcripts out of 26,107 annotated coding sequences were identified in WT CON_Phe_ and WT PD_Phe_ samples, respectively. 17,543 (67%) and 18,201 (70%) transcripts out of 26,107 annotated coding sequences were identified in WT CON_Stv_ and WT PD_Stv_ RNA-Seq libraries, respectively. 19,366 (74%) and 18,838 (72%) annotated transcripts were identified in the *csr-1* CON_Phe_ and *csr-1* PD_Phe_ samples, respectively. The *csr-1* CON_Stv_ and *csr-1* PD_Stv_ libraries contained 19,133 (73%) and 19,202 (74%) annotated transcripts, respectively. Based on the lower correlation of the CON_Phe_ replicate libraries (Fig S1), we examined their individual gene expression patterns in more detail. CON_Phe_ replicate 2 exhibited significant differences in gene expression from our previously published data for CON_Phe_ [10]; thus, it was excluded from the remainder of the analysis.

**qRT-PCR validation of seesaw genes**

qRT-PCR was done using RNA collected from three independent biological replicates from animals maintained at 20^o^C (wild-type N2, WM193 *csr-1*(*tm892)* IV; *neIs20* [*pie-1::3xFLAG::csr-1* + *unc-119*(+)], and HC196 *sid-1(qt9)*) V. To collect SS104 *glp-4(bn2)* I PD or CON adults, animals were routinely cultured at 15^o^C but shifted to the non-permissive temperature of 25^o^C from the embryonic stage until one-day-old adulthood to restrict the development of a functional germ line prior to sample collection for RNA extraction. Total RNA was treated with DNaseI (NEB) and processed with Superscript III or Superscript IV First Strand Synthesis Systems (Life Technologies) using oligo (dT) primers. Real-time PCR was done with iTaq Universal SYBR Green Supermix (BioRad). Primer sequences are listed in Table S8.

Thirty-three genes were selected that were: soma-enriched, CSR-1-targeted genes (*atp-4, f26h9.5, y67d2.3,* and *prx-19*); soma-enriched, non-CSR-1-targeted genes (*ins-19, mtl-1, f55b11.4, lys-7, ttr-5, fmi-1, hsp-16.41, f53a9.8, r12e2.15, spp-1, spp-2*, and *y51f10.7*); germline-enriched, CSR-1-targeted genes (*let-711, trr-1, cye-1, f45f2.10, isw-1, ifg-1, cbd-1, cgh-1, daz-1, gld-1, lin-41*, and *npp-9*); and germline-enriched, non-CSR-1-targeted genes (*cpb-1, noca-1, nspd-5, wago-2*, and *y48a6b.10*). The genes in the first three categories were chosen solely on their adequate abundance (average reads per kilobase of transcript per million mapped reads or rpkm of 147), as determined by RNA-Seq, to facilitate their detection in qRT-PCR assays. For the germline-enriched, non-CSR-1 targets, 4 out of 5 genes were not able to be validated using qRT-PCR (Figs S3D and S3E), likely due to their low abundance level (rpkm values between 0.55 to 74 with an average of 30) that is below the detection level to be accurately quantified by qRT-PCR. Normalization was set using 2 genes, *f28b4.3* and *y45f10d.4,* whose expression was unchanged in any control or dauer-inducing condition; or to the average of all qRT-PCR C_t_ values.

**Genomic clustering of CSR-1 targets**

The GLC algorithm defines clusters as the maximum number of adjacent genes (size) containing at least a minimum percentage of genes sharing the attribute (density). The algorithm iteratively cycles through all genes that possess the attribute, based on their physical order on each chromosome. A gene set between the first and last genes possessing the attribute on the chromosome is selected and it is determined if minimum size and density requirements are met. If so, the region is removed and the cluster is recorded, if not, the process continues iteratively analyzing the gene set between the first and next to last genes with the attribute, proceeding until either a cluster is identified or the gene set includes only the first gene, whereby it is removed and the process restarts at the next gene with the attribute. The process is repeated with the opposite linear orientation of gene order to account for directional differences, which are then reconciled by adjusting clusters to gene sets identified in either (inclusive) or only both (conservative) orientations. CSR-1 clusters were identified using the following parameters: the gene attribute was the list of CSR-1 targets, the minimum cluster size was three CSR-1 targeted genes, the minimum density was 0.66 and inclusive cluster reconciliation was used. Gene order was established based on the WormBase (WS235) annotations using only coding transcripts. Significance of clustering was compared to random expectations using non-parametric Monte Carlo simulations. Each simulation (10,000 iterations in total) involved randomizing gene order within the chromosome followed by genome-wide clustering. Significance of the prevalence of clusters, density of clusters and size of clusters were based on the percentage of simulations that showed values equal to or greater than those observed.

CSR-1 targets distributed in the genome as: 1,023 (chromosome I), 772 (II), 974 (III), 720 (IV), 617 (V), and 68 (X). CSR-1-targeted genes were found to be highly enriched in 507 genomic regions or clusters such that 121, 94, 115, 99, 77, and 1 clusters were located in chromosomes I, II, III, IV, V, and X, respectively. The largest cluster consisted of 77 genes (cluster_251 in chromosome III) spanning 357 kb; while the smallest clusters consisted of 3 genes (e.g. cluster_507 on the X chromosome). A detailed annotation of the CSR-1 clusters can be found in Table S6.

**GLD-1::GFP fluorescence, gonad area measurements, and germ cell counts**

**Preparation of CON_Phe_ and PD_Phe_ larva:** To collect populations of CON and PD larva under Phe conditions, we isolated crude dauer pheromone and performed dauer formation assays as described previously [79, 80]. Both dauer formation and control plates were made with Noble agar (BD Difco), and seeded with heat-killed *E. coli* OP50. To collect Phe-induced dauers, 10 reproductive BS1080 (*gld-1::gfp/flag*) hermaphrodites were allowed to lay eggs on assay plates containing 4.5 activity units of crude pheromone (1 activity unit = 33% percent dauer formation at 25ºC) until ~100 eggs were laid (approximately 2-6 hours). Dauers were distinguished from non-dauer worms based on body morphology, presence of alae, and lack of pharyngeal pumping [8]. Larva were allowed to remain in dauer for two days to prevent transient dauer entry before being transferred to seeded NGM plates for recovery. After approximately 6-12 hours, PD_Phe_ larva were imaged as described below. CON_Phe_ larva were collected by allowing reproductive BS1080 hermaphrodites to lay eggs as described above on control plates containing water instead of crude pheromone. Control larva were transferred from water plates to seeded NGM plates ~21-29 hours after egg laying, and allowed to develop until 40-48 hours post-egg laying before imaging. All plates were incubated at 20ºC.

**Preparation of CON_Stv_ and PD_Stv_ larva**: Stv-induced dauers were collected as described above, and allowed to develop for 8-14 hours on seeded NGM plates before imaging. For collection of CON_Stv_ larva, 60 to 100 *gld-1::gfp/flag* reproductive hermaphrodites were allowed to lay eggs on a 60 mm seeded NGM plate for 1 hour, after which they were removed. Hatched larva were grown continuously on the NGM plate for 40-44 hours post-egg laying before imaging.

**Imaging and analysis**: Larva were placed on a 2% agarose pad with 2 µl of 1 M sodium azide as an anesthetic for imaging using a Leica DM5500B microscope equipped with Hamamatsu camera C10600 ORCA-R^2^ and LAS AF software. Vulva morphology was determined for each larvae using Nomarski DIC microscopy at 630x as previously described [47, 48]. For animals exhibiting the vulva morphology characteristic of the L3, L4.1 and L4.4 larval stage, GFP fluorescence images were taken within the linear range under the same parameters for each animal over N ≥ 3 biologically independent trials.

Images were analyzed using ImageJ (NIH) by selecting the entire gonad arm with the polygon shape tool and recording measurements of integrated density, mean and maximum gray values, and area. Background fluorescent measurements were taken by selecting a 100 x 100 pixel area within the worm that was non-overlapping with the intestine or gonad. Corrected total cell fluorescence (CTCF) was calculated as “Integrated Density – (area * mean gray value of background)” [89]. Statistical significance between CON and PD samples was determined using Student’s *t*-test.

**Phenotypic analysis:** Proliferative cell analysis was performed using standard whole worm DAPI staining [81]. The live imaged larvae, described above, which had the L3, L4.1 and L4.4 vulva morphology were selected from the slide and fixed in cold 100% methanol for 10 minutes, DAPI stained for 15 minutes, and mounted in Vectashield (Vector Laboratories). The DAPI stained larvae were imaged using a Leica DM5500B microscope with the Hamamatsu camera controller C10600 ORCA-R2 and images recorded with the Leica LAS AF software.

The size of mitotic zone, transition zone, pachytene zone was determined based on the germ cell nuclear morphology [82]. The transition and pachytene zone were considered to start when at least two cells in a single row showed their specific morphology: crescent-shaped nuclei for transition zone and basket-shaped nuclei for pachytene zone. The total number of cell rows in the gonad was determined by adding the mitotic zone, transition zone and pachytene zone. Statistical significance between CON and PD samples was determined using Student’s *t*-test.

**Additional References**

88. Stiernagle T. Maintenance of C. elegans. WormBook. 2006:1-11. doi: 10.1895/wormbook.1.101.1. PubMed PMID: 18050451; PubMed Central PMCID: PMCPMC4781397.

89. McCloy RA, Rogers S, Caldon CE, Lorca T, Castro A, Burgess A. Partial inhibition of Cdk1 in G 2 phase overrides the SAC and decouples mitotic events. Cell Cycle. 2014;13(9):1400-12. doi: 10.4161/cc.28401. PMID: 24626186.
